# A hypovirulence-associated capsidless bi-segmented ssRNA mycovirus enhances melanin and microsclerotial production in a vascular phytopathogenic fungus

**DOI:** 10.1371/journal.ppat.1013348

**Published:** 2025-08-11

**Authors:** Jiamin Gao, Xuekun Zhang, Jichun Jia, Huang Huang, Jiasen Cheng, Yanping Fu, Xueqiong Xiao, Bo Li, Tao Chen, Xiao Yu, Longfu Zhu, Tom Hsiang, Daohong Jiang, Lili Zhang, Jiatao Xie

**Affiliations:** 1 State Key Laboratory Incubation Base for Conservation and Utilization of Bio-Resource in Tarim Basin, College of Life Science and Technology, Tarim University, Alar, China; 2 National Key Laboratory of Agricultural Microbiology, The Provincial Key Lab of Plant Pathology of Hubei Province, College of Plant Science and Technology, Huazhong Agricultural University, Wuhan, China; 3 Hubei Hongshan Laboratory, Wuhan, China; 4 Key Laboratory of Oasis Agricultural Pest Management and Plant Protection Resources Utilization, Xinjiang Uygur Autonomous Region, Shihezi University, Shihezi, China; 5 National Key Laboratory of Crop Genetic Improvement, College of Plant Science and Technology, Huazhong Agricultural University, Wuhan, China; 6 Environmental Sciences, University of Guelph, Guelph, Ontario, Canada; Okayama University, JAPAN

## Abstract

Mycoviruses are increasingly recognized for their multifaceted roles in fungal ecology, because of advances in understanding of their biology and molecular features. In this research, we identified and characterized two capsidless, bi-segmented positive-sense RNA mycoviruses: Verticillium dahliae ormycovirus 1 (VdOMV1) and VdOMV2, both of which infect *Verticillium dahliae*, a fungal pathogen causing vascular wilt of cotton. Phylogenetic analysis revealed that VdOMV1 and VdOMV2 cluster within the ormycovirus group, an evolutionary lineage unique to Riboviria. VdOMV2 may significantly enhanced *V. dahliae* melanin production and microsclerotial formation through regulating melanin synthesis-associated genes. This mediated the conversion from production of hyphae to microsclerotia, and enhanced *V. dahliae* survival under adverse abiotic stress conditions. Furthermore, VdOMV2 boosted the penetration ability of hyphae through cellophane membranes, while inhibiting the proliferation of *V. dahliae* hyphae within plants, and negatively modulated genes related to pathogenicity, possibly conferring hypovirulence. Enhancements in penetration and survival not only increase the efficacy of hypovirulent strains in overcoming environmental challenges, but also highlight the potential of VdOMV2-infected strains for managing Verticillium wilt in agricultural settings, thus representing an alternative mycovirus-based biocontrol approach for vascular fungal diseases.

## Author summary

Mycoviruses that confer hypovirulence to phytopathogenic fungi have been gaining attention for development of biocontrol agents. However, a major concern is poor fitness and survival of these mycovirus-mediated hypovirulent fungal strains, which limits their effectiveness in natural ecosystems. Therefore, it is crucial to develop mycoviruses-mediated hypovirulent strains that can thrive under various stress conditions. Here, two capsidless bi-segmented ssRNA mycoviruses, Verticillium dahliae ormycovirus 1 (VdOMV1) and VdOMV2, in an important plant vascular fungal pathogen *Verticillium dahliae* are molecularly characterized. VdOMV2 not only facilitates the conversion from hyphal to microsclerotial forms but also enhances the host survival in adverse abiotic stress conditions. Moreover, VdOMV2 improves the penetration ability of *V. dahliae* on cotton but confers hypovirulence on *V. dahliae*. This dual effect of hypovirulence and stress tolerance enhancement offers a novel mycovirus-based strategy to control vascular wilt diseases, including cotton Verticillium wilt.

## Introduction

Mycoviruses, which infect and replicate within fungal cells, are found across a diverse range of fungi, including those associated with plants, animals, humans, and diverse environmental samples. Advances in genome sequencing technology have led to a surge in the identification of mycoviruses, highlighting their widespread presence and importance in the virosphere. Mycoviruses have gained considerable attention for their potential as biocontrol agents, especially for chestnut blight caused by an ascomycetous fungus, *Cryphonectria parasitica* [1]. An RNA mycovirus, Cryphonectria hypovirus 1 (CHV1), was identified in a hypovirulent strain of *C. parasitica,* and was shown to significantly decrease fungal growth and conidial production. CHV1 was released into a natural *C. parasitica* population, resulting in successful control of chestnut blight in Europe [2], which highlighted the efficacy of mycoviruses in fungal disease management. Additionally, the DNA mycovirus, Sclerotinia sclerotiorum hypovirulence-associated DNA virus 1 (SsHADV1), confers hypovirulence on *Sclerotinia sclerotiorum*, causing white mold, and converts this harmful phytopathogenic fungus into a beneficial endophyte. This endophyte enhances the disease resistance of rapeseed and soybean plants to white mold, gray mold, and other fungal diseases [3,4], thereby providing a new strategy for utilizing mycoviruses as biocontrol agents (mycovirus-mediated plant vaccines) in agriculture [5].

*Verticillium dahliae* is a soil-borne vascular phytopathogenic fungus that causes Verticillium wilt, one of the most destructive fungal diseases [6]. In China, outbreaks of Verticillium wilt disease have resulted in reductions of more than 50% in both the planting area and fiber quality of cotton [7]. Furthermore, the proliferation of *V. dahliae* within the plant vascular tissues poses a significant challenge for chemical treatments that directly target the pathogen. Based on colony morphology and melanin biosynthesis, *V. dahliae* can be classified into three types: the microsclerotial colony types which produces melanin and microsclerotia; the hyphal colony types, which fails to produce melanin and microsclerotia but develops abundant aerial hyphae; and the intermediate colony types, which exhibits both partial melanin and microsclerotial production along with hyphal growth [8]. The microsclerotial colony types typically produce melanized microsclerotia and melanin, which enhance resistance to abiotic stresses and enable survival in the soil for over a decade [9,10]. However, under certain instances, the hyphal colony type may exhibit increased virulence relative to the microsclerotial colony types [8,11]. To date, only four mycoviruses have been identified in *V. dahliae*, including three mycoviruses within *Chrysoviridae*, *Partitiviridae*, and *Botourmiaviridae*, as well as an unassigned mycovirus phylogenetically related to members of *Tombusviridae* [12–15]. However, the diversity of mycoviruses infecting *V. dahliae* remains relatively unexplored.

Ormycoviruses are positive-sense RNA (+ssRNA) viruses with two genomic segments. The large segment encodes an RNA-dependent RNA polymerase (RdRp), and the small segment encodes a hypothetical protein of unknown function. Ormycoviruses were first identified in a biological sample from the yeast *Starmerella bacillaris*, while subsequent studies detected ormycovirus-related sequences in powdery mildew lesions on vegetable crops and grapevines through high-throughput RNA sequencing [16]. They were later molecularly characterized in *Trichoderma* spp*.* and *Hortiboletus rubellus* [17,18]. At present, the 23 reported ormycoviruses are categorized into three distinct groups: *Alphaormycovirus*, *Betaormycovirus,* and *Gammaormycovirus* [16–19]. These ormycoviruses are currently placed under *unclassified Riboviria* within *Riboviria* and represent a group of viruses belonging to *Ormycoviridae*.

In this study, we used virome analysis to identify two capsidless +ssRNA mycoviruses, Verticillium dahliae ormycovirus 1 (VdOMV1) and VdOMV2, from *V. dahliae*. We further investigated their biological functions in inducing hypovirulent phenotypes and converting from hyphal colony types to microsclerotial colony types of *V. dahliae*, as well as their potential for biocontrol of Verticillium wilt in cotton. Finally, we explored the mechanisms underlying hypovirulence and conversion of hyphal colony types induced by VdOMV2 infection.

## Results

### Identification of three mycoviruses infecting *V. dahliae*

**Fig 1:**
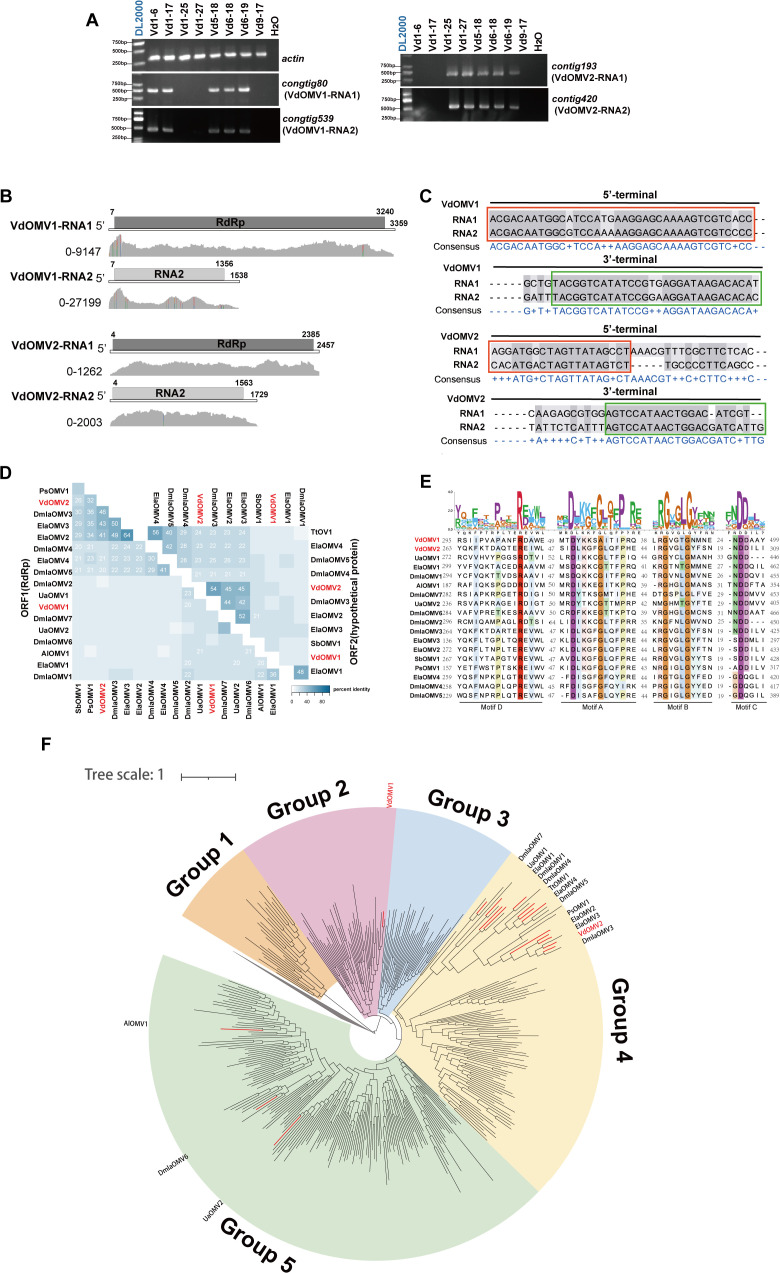
Molecular characterization of VdOMV1 and VdOMV2. *(A)* RT-PCR confirmation of *de novo* assembled contigs from strains generated by Illumina sequencing. *(B)* Schematic representation of the genomic organization of VdOMV1 and VdOMV2. Grey peaks represent coverage obtained by mapping the ribose-depleted RNA reads to the viral genome. *(C)* Multiple alignments of the 5′- and 3′-terminal regions of VdOMV1 and VdOMV2 genomes. The red box represents the conserved nucleotide sequence at the 5′-terminal regions, the green box represents the conserved nucleotide sequence at the 3’-terminal regions, and the background of conserved bases is colored in gray. *(D)* The percent identity matrix constructed based on ormycovirus RdRps and hypothetical proteins. Identity values >20% are shown, VdOMV1 and VdOMV2 are highlighted with red font. *(E)* Multiple alignments of ormycovirus RdRps. VdOMV1 and VdOMV2 are marked in red. The background of conserved amino acids is colored, and different colors represent amino acids with different properties. The number represents the amino acids shared among the conserved regions. The four conserved motifs in RdRps of ormycovirus are indicated above the sequences. *(F)* The maximum-likelihood phylogenetic tree was constructed using representative sequences from Supergroup024 and established ormycoviruses. Supergroup026 and Qin-Yue lineages served as outgroups, based on prior taxonomic classification by Hou et al. [20]. The ormycoviruses are divided into five major clades (Group 1 to Group 5), with VdOMV1 and VdOMV2 highlighted in red font. The scale bar (tree scale = 1) indicates the number of substitutions per site.

We constructed nine metatranscriptomic sequencing libraries (VdA1-VdA9) of 381 *V. dahliae* strains isolated from diseased cotton stalks, and conducted metatranscriptome analysis to explore mycovirus diversity in *V. dahliae.* The reads were assembled in 7766 contigs. Only 14 virus-associated contigs (>500 nt) were detected in four libraries (VdA1, VdA5, VdA6, and VdA9). These contigs were further characterized, and found to represent five distinct viral sequences associated with three mycoviruses, namely, VdMoV1 (Verticillium dahliae magoulivirus 1), VdOMV1, and VdOMV2. Subsequent RT-PCR with primers specific for these three mycoviruses confirmed their presence in eight of the 381 *V. dahliae* strains (Fig 1A and S1 Table).

To determine whether virus-infected strains harbored other undetected mycoviruses, we conducted metatranscriptomic analysis again of strain Vd1–6 co-infected by VdMoV1 and VdOMV1, as well as strain Vd1–25 infected by VdOMV2. Five virus-associated contigs (contig90, contig110, contig202, contig204, and contig110) completely matching those found in the previously constructed nine libraries (VdA1-VdA9) were detected in strains Vd1–6 and Vd1–25, with no other sequences being associated with known viruses. Contig90 matched the RdRp of Downy mildew lesion-associated ormycovirus 2 (DmlaOMV2), and contig110 encoded a protein identical to the previously reported VdMoV1. Additionally, the putative protein encoded by contig94 shared low identity with RdRp of DmlaOMV3, while contig204 shared homology with a hypothetical protein of DmlaOMV3. Notably, contig202 lacked similarity to proteins in the NR (Non-Redundant Protein Sequence) database of NCBI. RT-PCR analysis using specific primers confirmed the presence of contig90, contig202, and VdMoV1 (contig110) in strain Vd1–6, whereas strain Vd1–25 harbored both contig94 and contig204 (S1 Fig).

Based on detailed analysis, contig202 and contig90 were found to belong to Verticillium dahliae ormycovirus 1 (VdOMV1) infecting strain Vd1–6, while contig204 and contig94 were partial genomes of another ormycovirus, VdOMV2, in strain Vd1–25.

### VdOMV1 and VdOMV2 with positive-sense RNA genomes contain two RNA segments

Next, the complete genomes of VdOMV1 and VdOMV2 were determined. They harbored bipartite +ssRNA genomes (RNA1 and RNA2). The genomes of VdOMV1 and VdOMV2 showed even coverage when the clean reads were aligned, suggesting a consistent sampling of base positions (Fig 1B). VdOMV1 RNA1 consisted of 3359 nucleotides (nt) and contained a single open reading frame (ORF1, nt positions 7–3240) encoding a putative RdRp, while RNA2 of VdOMV1 encoded a 51.3 kDa hypothetical protein (ORF2, nt positions 7–1356) (Fig 1B). VdOMV2 had a single ORF (ORF1, nt positions 4–2385) in RNA1, encoding a putative RdRp; and RNA2 of VdOMV2 encoded a 57.5 kDa hypothetical protein (ORF2, nt positions 4–1563). Notably, the 5′ -terminal sequences of RNA1 and RNA2 from VdOMV1 shared about 40-nt sequence, and the 3′ -terminal sequences within 40 nt were highly conservative (Fig 1C). The RNA1 and RNA2 of VdOMV2 were identical for around 20 nt in the 5′ and 3′ terminal sequences (Fig 1C).

To investigate the relationship between VdOMV1, VdOMV2, and other known ormycoviruses, we constructed an identity matrix (Fig 1D). RdRps of VdOMV1 and VdOMV2 shared less than 55% identity with those of previously reported ormycoviruses. Furthermore, the hypothetical protein encoded by RNA2 of VdOMV2 exhibited less than 50% identity with known ormycoviruses, while the hypothetical protein of VdOMV1 did not share any identity with any known proteins. Notably, the RdRp of VdOMV1 exhibited only 11% identity with that of VdOMV2, while the hypothetical proteins encoded by VdOMV1 and VdOMV2 shared only 10% identity. The RdRps of VdOMV1 and VdOMV2 contained four conserved motifs, including the NDD catalytic triad in motif C (Fig 1E).

To investigate the phylogenetic relationships of VdOMV1 and VdOMV2 within the family *Ormycoviridae*, we searched against the database published by Hou et al [20]. using an HMM method for putative ormycovirus sequences [20]. Around 350 ormycovirus sequences were identified with a NDD motif. Notably, these ormycovirues were classified within Supergroup024 in the Hou et al. [20] study. A maximum-likelihood phylogenetic analysis was performed using representative sequences from Supergroup024 and established ormycoviruses [20]. The results revealed that ormycoviruses could be classified into five major clades (Group1–Group5) (Fig 1F). VdOMV1 clustered within Group2, while VdOMV2 formed part of the novel Group4 lineage. Notably, three clades demonstrated congruence with existing taxonomic divisions: Group2 with *Alphaormycovirus*, Group3 with *Betaormycovirus*, and Group5 with *Gammaormycovirus* (phylogenetic tree containing all virus sequence names is in S2 Fig) [16]. However, the identification of two previously unrecognized clades (Group1 and Group4), comprised of multiple divergent ormycovirus-related sequences, suggested substantial hidden diversity within this viral family. This expanded phylogenetic framework indicated that current classification schemes significantly underestimate the genetic heterogeneity and evolutionary complexity of ormycoviruses.

### VdOMV1 and VdOMV2 are capsidless viruses

To investigate whether VdOMV2 encodes a coat protein, we used VdPV1 (Verticillium dahliae partitivirus 1) with spherical virions as a positive control [13]. VdPV1 was successfully introduced into strain Vd1–25 by anastomosis (S3A Fig). The introduction of VdPV1 did not reduce the accumulation of VdOMV2 (S4 Fig); on the contrary, occasional increases in VdOMV2 were observed. We attempted to purify virions multiple times from strains Vd1–25 and Vd1–25-P (S3B Fig). Transmission electron microscopy (TEM) observations revealed that only strain Vd1–25-P contained spherical virions with diameters of approximately 20 nm (S3C Fig). However, similar virions were not observed in strain Vd1–25. SDS-PAGE analysis of virions purified from strain Vd1–25-P exhibited a single protein band (S3D Fig). LC-MS/MS analysis of this band revealed that six peptides accurately matched the coat protein of VdPV1, and no peptides were associated with VdOMV2 (S3E Fig). Therefore, we speculated that VdOMV2 was most likely unable to form virions.

**Fig 2:**
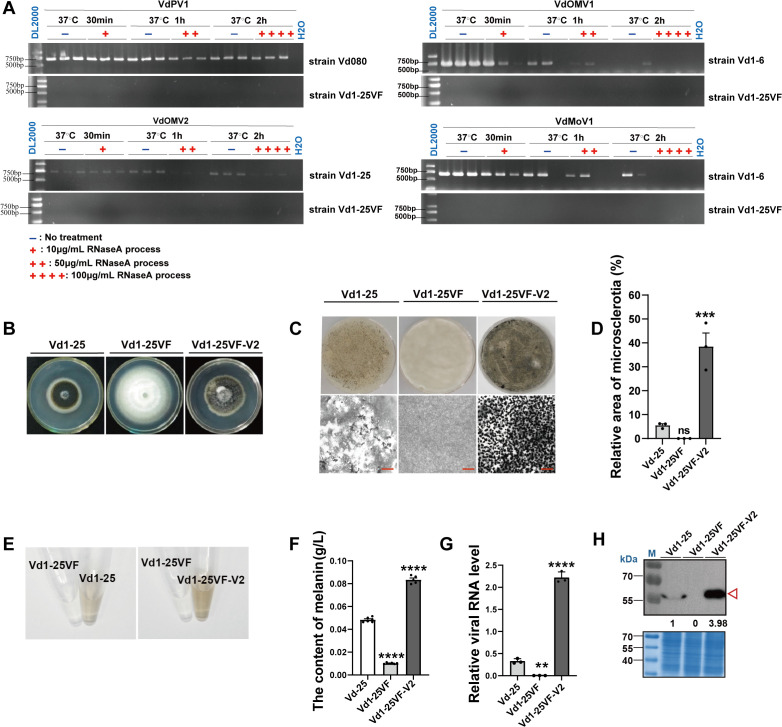
VdOMV2 infection change biological features of *V. dahliae.* ***(A)***. Susceptibility of viral RNAs to RNase **A.** Electrophorogram of RT-PCR with mycovirus-specific primers. The three strains are VdPV1-infected strain Vd1-25-P, VdOMV1 and VdMoV1-co-infected strain Vd1-6 and VdOMV2-infected strain Vd1-25. “+” denotes samples treated with RNase A, while “-” denotes untreated samples. ***(B)*** The colony morphology of VdOMV2-infected strains Vd1-25 and Vd1-25VF-2, and virus-free strain Vd1-25VF on the PDA (25°C, 15 dpi). ***(C)*** The conidia of VdOMV2-infected and -free strains were cultured on cellophane overlying PDA to observe microsclerotial production (25°C, 15 dpi), and photographed with a camera *(upper panel)* and a stereoscope *(lower panel)*. Scale bar = 400 μm. ***(D)*** Relative area of microsclerotia produced by VdOMV2-infected strains Vd1-25 and Vd1-25VF-V2. Relative area of microsclerotial production was evaluated by the density of gray color using ImageJ scanning. ***(E)*** Melanin was extracted from the VdOMV2-infected and -free strains to measure melanin production. ***(F)*** Melanin content of VdOMV2-infected and -free strains. ***(G)*** VdOMV2 levels in strains Vd1-25VF-V2 and Vd1-25 were detected by qRT-PCR. ***(H)*** Western blot analysis showing VdOMV2 protein levels in strains Vd1-25VF-V2 and Vd1-25, quantification of the protein levels was evaluated by the density of gray color using ImageJ scanning, mark below the PVDF membrane and Vd1-25 was set to 1 *(upper panel)*. The SDS-PAGE gels of three strains were stained with Coomassie Brilliant Blue to show the protein loading *(lower panel)*. The data were analyzed using Student’s t-test for ***(G)***, one-way ANOVA for ***(D)***, and two-way ANOVA for ***(F)***. Statistical significance is indicated as follows: ns*P > *0.05; *P < 0.05; **0.001* < P < *0.01; ***0.0001* < P < *0.001; ****P < 0.0001; and error bars represent standard deviation (SD).

To confirm the capsidless nature of VdOMV1 and VdOMV2, mycelial homogenates from strains Vd1–6, Vd1–25, and Vd080 were treated with RNase A at varying concentrations (10–100 µg/mL) and durations (30 min–2 h). RT-PCR showed that VdPV1 (capsid-protected control) remained detectable under all conditions, while VdMoV1 (capsidless control), VdOMV1, and VdOMV2 RNAs were degraded at higher RNase A concentrations or with longer exposures (Fig 2A). VdOMV2 RNA was not detected after 1 h at 50 µg/mL, indicating high susceptibility to degradation (Fig 2A). These results confirmed that VdOMV1 and VdOMV2 lack protective capsids and cannot form virions.

### VdOMV2 infection results in conversion from hyphal- to microsclerotial colony types in *V. dahliae*

Using a combination of mycovirus elimination techniques, including the antiviral drug ribavirin, single conidia isolation, and hyphal tip isolation, we successfully obtained two ormycovirus-free strains: Vd1–6VF and Vd1–25VF (S5A and S5B Fig). We then compared the colony morphology and virulence between ormycovirus-free and -infected strains. VdOMV1 infection primarily affected the production of melanin and microsclerotia, without significantly altering the pathogenicity of *V. dahliae* (S5C and S5D Fig). Whereas VdOMV2 infection could be related to hypovirulence, notably impairing melanin biosynthesis and the melanization of microsclerotia (S5B and S5C Fig). Therefore, we focused on VdOMV2 in our subsequent experiments. To further confirm whether VdOMV2 was associated with the observed phenotypic changes, we introduced VdOMV2 into the mycovirus-free strain Vd1–25VF via hyphal fusion, creating a new VdOMV2-infected strain, Vd1–25VF-V2 (S6A Fig). The horizontal transmission efficiency of VdOMV2 was 96% (23/24), and 74% (16/23) into the newly infected strains, and they remarkably exhibited similar morphology to strain Vd1–25 (S6A and S6B Fig).

Compared with Vd1–25VF, the phenotypes of both Vd1–25 and Vd1–25VF-V2 showed significantly enhanced melanin biosynthesis and melanized microsclerotia (Fig 2B). Vd 1–25VF-V2 produced approximately 3-fold more microsclerotia (422 microsclerotia) than strain Vd1–25 (116 microsclerotia) (Fig 2C and 2D), and the production of melanin on PDA was 2-fold higher for Vd1–25VF-V2 at 15 days post-inoculation (dpi) (Fig 2E and 2F). This was consistent with VdOMV2 accumulation, as qRT-PCR analysis showed approximately 6-fold higher amounts in strain Vd1–25VF-V2 than in strain Vd1–25 (Fig 2G), and Western blot analysis revealed that the hypothetical protein content of VdOMV2 in strain Vd1–25VF-V2 was approximately 4-fold higher than in strain Vd1–25(Fig 2H). Therefore, we speculated that VdOMV2 positively regulates melanized microsclerotia and melanin biosynthesis in *V. dahliae*, and is likely responsible for the phenotypic conversion from the hyphal-type to the microsclerotial colony types in *V. dahliae*.

### VdOMV2 enhances *V. dahliae* tolerance to abiotic stress conditions

**Fig 3:**
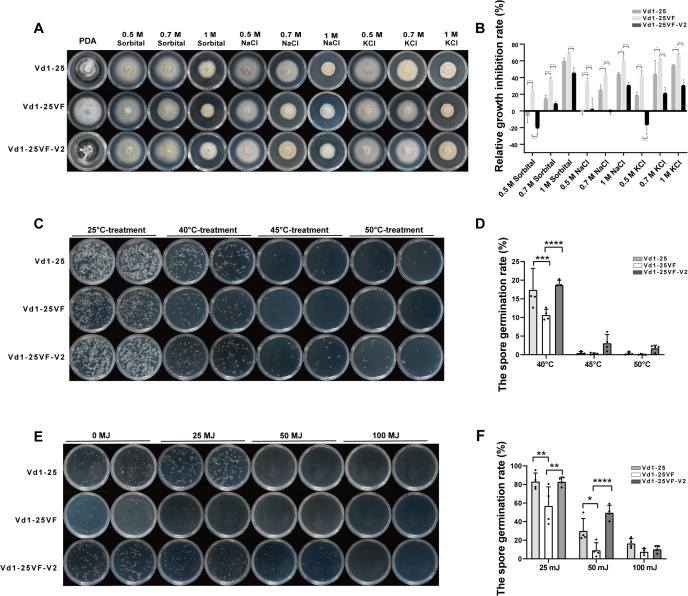
VdOMV2 infection enhances the survival of *V. dahliae* under adverse abiotic stress conditions. ***(A)*** Colony morphology of strains Vd1-25, Vd1-25VF and Vd1-25VF-V2 on PDA supplemented with different abiotic stresses. ***(B)*** Relative growth inhibition of *V. dahliae* colonies by each stress treatment. *(C)* and ***(D)*** The conidia germination of strains Vd1-25, Vd1-25VF and Vd1-25VF-V2 at 3 dpi on PDA following different heat treatment for 10 min. *(E)* and ***(F)*** The conidial germination of strains Vd1-25, Vd1-25VF and Vd1-25VF-V2 at 3 dpi on PDA following different UV irradiation treatment. Each treatment was performed in triplicate. The data were analyzed by two-way ANOVA. Statistical significance is indicated as follows: ns *P > *0.05; *P < 0.05; **0.001* < P < *0.01; ****P < 0.0001.

To evaluate the role of VdOMV2 in environmental stress adaptation, we compared physiological responses between isogenic strains: wild-type Vd1–25 (VdOMV2-infected), cured strain Vd1–25VF (virus-free), and re-infected control Vd1–25VF-V2. The growth rates or conidial germination rates were quantified on PDA under abiotic stress conditions. Osmotic stress assays revealed significant growth impairment in the cured strain (Vd1–25VF) compared to both virally-infected lines when challenged with sorbitol, NaCl, or KCl (Fig 3A and 3B), suggesting that VdOMV2 confers enhanced osmoregulatory capacity. Thermotolerance showed strain-specific responses: all tested strains exhibited no significant differences in conidial germination efficiency at 25°C. Following heat shock at 40°C, 45°C, or 50°C for 10 min, the germination of Vd1–25VF conidia was significantly higher than that of Vd1–25 and Vd1–25VF-V2, but no significant differences in germination rates were observed among the three strains when the temperature was increased to 45°C or 50°C. UV susceptibility assays demonstrated dose-dependent inhibition, with cured conidia showing 2.1- and 3.4-fold greater sensitivity at 25/50 mJ/cm² exposures compared to virus-infected strains (p < 0.001). Notably, 100 mJ/cm² UV irradiation achieved near-complete inhibition of germination across all phenotypes (Fig 3E and 3F). Overall, these results indicated that VdOMV2 significantly enhances *V. dahlia*e tolerance under adverse abiotic stress conditions.

### VdOMV2 confers hypovirulence to *V. dahliae*

**Fig 4:**
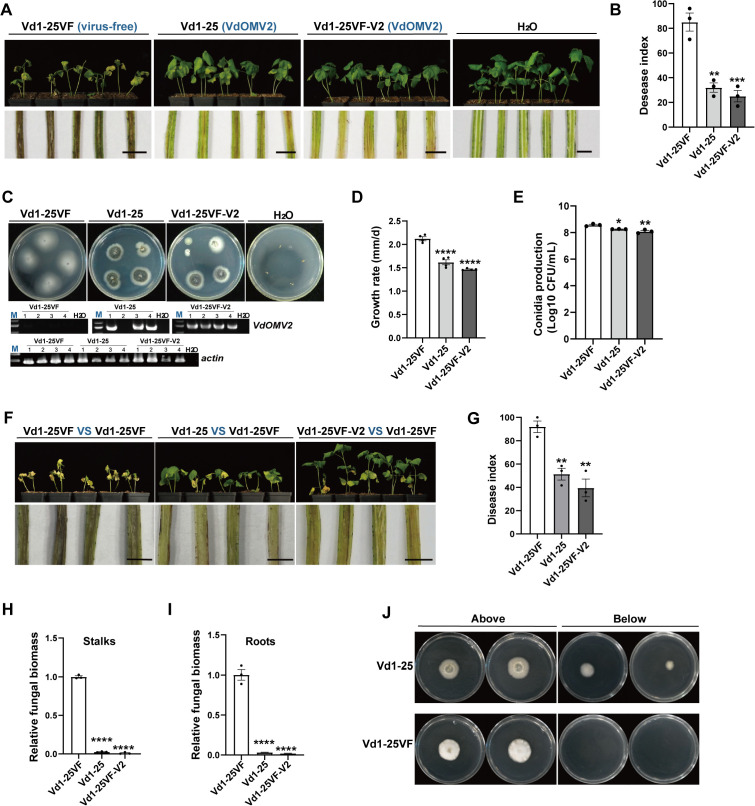
VdOMV2 confers hypovirulence in *V. dahliae.* *(A)* and ***(B)*** Virulence assays of VdOMV2-free strain Vd1-25VF and -infected strain Vd1-25 and Vd1-25VF-V2 on cotton seedlings. 10 days-old cotton seedlings were inoculated with 10^8^/mL conidia of fungal strains, and the Verticillium wilt symptom *(A, upper panel)* were photographed at 45 dpi on cotton with additional phenotype of vascular discoloration *(A, lower panel)*, respectively. ***(C)*** The colony morphology of strains isolated from the cotton stalks inoculated by fungal strains as shown in Fig 3A *(upper panel)*, and VdOMV2 confirmation in re-isolated strains by RT-PCR *(lower panel)*. ***(D)*** The growth rate and *(E)* conidial production of strains Vd1-25, Vd1-25VF, and Vd1-25VF-V2. *(F-I)* Biocontrol assay of VdOMV2 on the live cotton seedlings. The cotton seedlings were pre-inoculated with strains Vd1-25, Vd1-25VF-V2, or Vd1-25VF, and then then re-inoculated after 5 days using virulent strain Vd1-25VF. TheVerticillium wilt symptom *(F, upper panel)* were photographed at 45 dpi on cotton as well as vascular discoloration *(F, lower panel)*. ***(G)*** The disease index of cotton Verticillium wilt was calculated at 45 dpi. Fungal biomass in cotton stalks *(H)* and roots *(I)* were assayed by qPCR. ***(J)*** Strains Vd1-25 and Vd1-25VF were cultured for 3 days on cellophane overlying PDA (above) and then three days after removal of the cellophane (below). Scale bar = 0.5 cm. Data were analyzed using one-way ANOVA, *P < 0.05, **0.001* < P < *0.01, ***0.0001* < P < *0.001; ****P < 0.0001.

The disease index (0 = low, 100 = high) was significantly lower in VdOMV2-infected strains (32 for Vd1–25 and 25 for Vd1–25VF-V2) than in strain Vd1–25VF that had a disease index of 85 (Fig 4A and 4B); correspondingly, the rates of diseased plants were 83% for Vd1–25, 44% for Vd1–25VF-V2, and 100% for Vd1–25VF (Fig 4A). Notably, although vascular discoloration was observed in cotton plants inoculated with strain Vd1–25VF-V2, the overall plant phenotype, including the leaves, appeared healthier compared to those of plants inoculated with strain Vd1–25 (Fig 4A). Furthermore, *V. dahliae* re-isolated from stalks treated with strains Vd1–25 and Vd1–25VF-V2 exhibited colony morphology similar to that of VdOMV2-infected strains, and all isolates tested positive for VdOMV2 (Fig 4C). The growth rates of re-isolated strains were not significantly different from those of strains Vd1–25 or Vd1–25VF-V2, but were significantly slower than that of strain Vd1–25VF (Fig 4D). The number of conidia produced by strain Vd1–25VF-V2 was significantly less than strain Vd1–25, and both were fewer than strain Vd1–25VF (Fig 4E). Importantly, the inverse correlation between VdOMV2 accumulation (Fig 2F and 2G) and pathogenicity among the three strains (Fig 4) suggested that VdOMV2 may be associated with hypovirulence in *V. dahliae*.

To further assess the biocontrol potential of VdOMV2 against Verticillium wilt, we performed virulence assays with three treatment groups: pre-inoculation with strains Vd1–25, Vd1–25-V2, or Vd1–25VF, followed by inoculation with strain Vd1–25VF to the three treatment groups after a five-day interval (S7 Fig). The disease index for cotton treated with strain Vd1–25VF was 92.6, and the corresponding diseased cotton rate was 95%. For cotton separately treated with strains Vd1–25 and Vd1–25VF-V2, the disease indices were 46.3 and 38.8, respectively, with disease rates of 75% and 80%, respectively (Fig 4F and 4G). The biocontrol efficiencies of strains Vd1–25 and Vd1–25VF-V2 were 50% and 58.1%, respectively. Moreover, compared to cotton treated with strain Vd1–25VF, the fungal biomass of *V. dahliae* was significantly lower in the stalks, and roots of cotton plants treated with VdOMV2-infected strains (Fig 4H and 4I). Specifically, cotton stalks treated with strain Vd1–25 exhibited a 37-fold reduction in estimated fungal biomass within stalks, and a 30-fold reduction in the roots. Those treated with strain Vd1–25VF-V2 showed a 100-fold reduction in estimated fungal biomass in the stalks and a 50-fold reduction in the roots (Fig 4H and 4I). Therefore, cotton plants treated with VdOMV2-infected strains showed significantly reduced Verticillium wilt symptoms, including foliar wilt and vascular discoloration, because of lower fungal proliferation within the plants.

We also assessed the penetration ability of strains Vd1–25 and Vd1–25VF *in vitro* using cellophane film. Mycelial plugs of Vd1–25 and Vd1–25VF were inoculated onto cellophane overlying PDA, and after incubation at 25 °C for 3 days, the film with the mycelial plugs were removed, and the PDA was observed for the next three days for fungal growth. This method has been widely used for assessing the penetration ability of *V. dahliae* [21–25]*.* Only Vd1–25 was observed to grow on the PDA after the cellophane was removed, whereas no growth of Vd1–25VF was observed (Fig 4J). Therefore, VdOMV2 infection may enhance the penetration ability of *V. dahliae*.

### VdOMV2 evokes transcriptional rewiring and regulates key genes associated with melanogenesis and pathogenicity in *V. dahliae*

**Fig 5:**
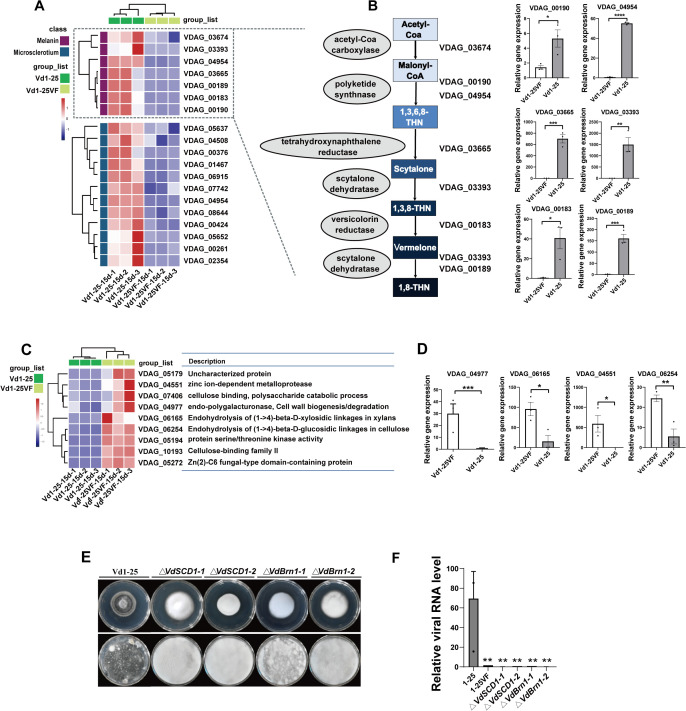
VdOMV2 evokes transcriptional rewiring and regulates melanin biosynthesis-related genes in *V. dahliae.* ***(A)*** Expression profiles of identified genes for melanin and microsclerotia in *V. dahliae*. Red means a high expression level and purple indicates a low expression level. ***(B)*** The pathways and key genes involved in melanin formation and the expression levels of these key genes were detected using qRT-PCR. ***(C)*** Expression profiles of identified CWDE genes in *V. dahliae*. The redder the color, the higher the gene expression level, and the more purple the color, the lower the expression level. The table on the right describes the corresponding genes. ***(D)*** The expression of CWDEs were detected by qRT-PCR in strains Vd1-25 and Vd1-25VF. There were biological and technical replicates for each qRT-PCR. ***(E)*** Colony morphology of strain Vd1-25, and its *VdSCD1* (VDAG_03393) and *VdBrn1* (VDAG_03665) deletion mutants on PDA (25°C, 15 dpi) (*upper panel).* The conidia of strain Vd1-25, and its *VdSCD1* and *VdBrn1* deletion mutants were cultured on cellophane overlying PDA to observe microsclerotia *(lower panel)*. ***(F)*** VdOMV2 levels in strains Vd1-25 and the deletion mutants were detected using qRT-PCR. Date were analyzed using Student’s t-test, ****P < 0.0001; ***0.0001* < P < *0.001; **0.001* < P < *0.01; *P < 0.05.

We conducted comparative transcriptomic analyses between strains Vd1–25 and Vd1–25VF (Figs. S8A–S8B and S5A–S5D). Compared to strain Vd1–25VF, 1389 differentially expressed genes (DEGs) were up-regulated, and 646 DEGs were down-regulated in strain Vd1–25. Enrichment analysis revealed that VdOMV2 may play a role in regulating host metabolism and intercellular signaling in *V. dahliae*. We targeted analysis of pathways associated with melanin and microsclerotial formation*.* Seven key genes are involved in the melanogenesis pathway, which converts from acetyl-CoA to 1,8-dihydroxynaphthalene [9], and we observed that six of these genes exhibited significantly up-regulated expression in Vd1–25 (Fig 5B). These six genes encode polyketide synthase (VDAG_00190), melanin biosynthesis protein Ayg1 (VDAG_04954), hydroxynaphthalene reductase (VDAG_03665, VdBrn1), scytalone dehydratase (VDAG_03393, VdSCD1), versicolorin reductase (VDAG_00183), and laccase (VDAG_00189) (Fig 5B). In addition, strain Vd1–25 showed upregulation of 12 reported genes linked to melanized microsclerotia (Fig 5A). These genes encode chitin synthase D (VDAG_00376) [26], glucose-repressible protein (VDAG_01467) [27], adenylate cyclase (VDAG_04508) [28], hypothetical protein (VDAG_05652) [21], IDI-3 protein (VDAG_00261) [9,27], mitogen-activated protein kinase HOG1 (VDAG_02354) [29], two hypothetical proteins (VDAG_00424 and VDAG_07742) [30,31], MFS gliotoxin efflux transporter GliA (VDAG_05637) [32], HPt domain-containing protein (VDAG_06915) [33], pigment biosynthesis protein Ayg1 (VDAG_04954) [24], and C2H2 type zinc finger domain-containing protein (VDAG_08644) (Fig 5A) [34]. Therefore, VdOMV2 can promote melanin biosynthesis and microsclerotial melanization in *V. dahliae* through the regulation of associated genes, potentially facilitating hyphal to microsclerotialcolony conversion.

Cell wall degrading enzymes (CWDEs) are produced by *V. dahliae* to degrade plant cell wall barriers and xylem cell walls, thereby facilitating the colonization of host plant cells [35–37]. In transcriptomic analysis, we focused on identifying DEGs associated with CWDEs. There was significant downregulation of genes encoding CWDEs in strain Vd1–25, including Chitin-binding lysin (VDAG_05179) [38], allergen Asp F2-like protein (VDAG_04551, VdASPF2) [39,40], endoglucanase-1 (VDAG_07406), endopolygalacturonase (VDAG_04977) [41], endo-1,4-beta-xylanase A (VDAG_06165) [42], endoglucanase (VDAG_06254) [27], Zn(2)-C6 fungal-type domain-containing protein (VDAG_05272) [43], EKC/KEOPS complex subunit BUD32 (VDAG_05194) [44], as well as cellulose-binding family II (VDAG_10193) (Fig 5C and 5D) [45]. The observed downregulation of CWDE genes in VdOMV2-infected strains suggested a potential alteration in extracellular enzymatic activity, though the functional implications require further investigation.

To further evaluate the biological impact of this transcriptional modulation, we conducted indirect fungal biomass assays using qPCR in cotton stems and roots infected with the VdOMV2-infected strain. The results demonstrated a significant reduction in estimated fungal biomass, indicating that VdOMV2 may attenuate virulence by limiting fungal proliferation within host tissues (Fig 4H and 4I). This finding is consistent with the transcriptomic data, reinforcing the hypothesis that VdOMV2 contributes to reduced virulence by downregulating key CWDE genes, thereby constraining pathogen dissemination in cotton plants.

Comparative transcriptomic analyses revealed that scytalone dehydratase (VdSCD1, VDAG_03393) and hydroxynaphthalene reductase (VdBrn1, VDAG_03665) were significantly upregulated in response to VdOMV2 infection (Fig 5A), whereas the allergen Asp F2-like protein (VdASPF2, VDAG_04551) exhibited downregulated expression (Fig 5C). *VdASPF2* has been demonstrated to be associated with the pathogenicity of *V. dahliae*, and its deletion results in reduced virulence towards cotton [40]. The upregulated *VdSCD1* and *VdBrn1* were predicted to play key roles in melanin biosynthesis [46,47].

To further investigate their functional roles in morphological development, colony characteristics, and melanin biosynthesis of *V. dahliae*, we constructed deletion mutants of *VdSCD1* and *VdBrn1* of strain Vd1–25 (S9A and S9B Fig). Phenotypic analysis revealed that neither the *ΔVdSCD1* nor *ΔVdBrn1* mutants were capable of synthesizing melanin or forming microsclerotia at 15 dpi (Fig 5E), displaying phenotypes identical to strain Vd1–25VF (S10A and S10B Fig). Additionally, quantification of VdOMV2 accumulation in the deletion mutants revealed a significant reduction in viral accumulation levels, which closely resembled those observed in Vd1–25VF (Fig 5F).

Collectively, these findings indicated that *VdSCD1* and *VdBrn1* were not only essential for melanin biosynthesis and microsclerotial formation but also played a crucial role in maintaining VdOMV2 accumulation. The observed reduction in VdOMV2 levels upon deletion of these genes suggested a potential link between melanin biosynthesis and VdOMV2 expression or stability, implicating *VdSCD1* and *VdBrn1* in the regulation of viral homeostasis or replication.

## Discussion

In this study, two novel capsidless bi-segmented +ssRNA mycoviruses, namely VdMOV1 and VdOMV2, were identified and characterized from *V. dahliae*. They were all identified as ormycoviruses based on genomic organization (two RNA segments) and phylogenetic analysis of RdRp sequences. VdOMV2 played a role in conversion from the hyphae to microsclerotia colony types, and also exhibited promising potential as a biocontrol agent for managing Verticillium wilt in cotton. These findings expand our understanding of ormycovirus biology and provide a case study on mycovirus-mediated biocontrol against vascular fungal diseases.

Three mycoviruses were finally identified in the virome of 381 strains of *V. dahliae*, indicating a low mycovirus diversity in this fungal species. To date, only six mycoviruses have been characterized in *V. dahliae* [12–15], including VdOMV1 and VdOMV2. The low diversity of mycoviruses in *V. dahliae* isolated from cotton could be attributed to several potential factors, although these hypotheses remain speculative and require further experimental validation. One possibility is that the pathogen’s specialized infection of the cotton vascular system, coupled with the formation of dormant microsclerotia, may limit the opportunity for mycovirus transmission and diversification [44]. Additionally, cotton (*Gossypium* spp.) is rich in flavonol compounds, including gossypetin (GOP), gossypin, and gossypol [48–51], which exhibit antiviral activity against ssRNA viruses [52,53], potentially further reducing the prevalence of mycoviruses in *V. dahliae*. Future work should test whether purified flavonols suppress VdOMV2 replication *in vitro* or *in planta.*

To date, 23 ormycoviruses have been identified, but their impacts on fungal phenotypes have not been thoroughly investigated [16–19]. Our comprehensive maximum-likelihood phylogenetic analysis, incorporating all currently recognized ormycovirus sequences along with newly identified variants, revealed a more complex evolutionary landscape than previously appreciated. The resulting phylogeny not only corroborated the established classification system (including *Alpha-*, *Beta-*, and *Gammaormycovirus* genera), but also identified two previously unrecognized monophyletic groups (Group1 and Group4). Particularly noteworthy was the robust clustering of VdOMV2 within the novel Group4 clade, which exhibited substantial genetic divergence from characterized ormycovirus lineages. These findings, combined with the identification of unclassified but phylogenetically distinct sequences (e.g., Supergroup024), suggested that current taxonomic frameworks capture only a fraction of ormycovirus diversity. The expanded phylogenetic diversity revealed by this study underscores the urgent need for systematic investigations into the molecular characteristics and host interactions of these newly identified ormycovirus lineages. In this study, we demonstrated that VdOMV2 could be associated with hypovirulence in *V. dahliae*, thereby significantly expanding the biological significance of ormycoviruses, and also providing a potential biocontrol agent against Verticillium wilt of cotton. However, definitive proof of hypovirulence would require infection assays using pure VdOMV2 or its infectious clones. Currently, construction of an infectious clone for VdOMV2 remains technically unfeasible at present due to its bisegmented RNA genome and the lack of viral particles for reverse genetics approaches.

Current control measures for vascular fungal diseases primarily rely on the chemical suppression of vascular-inhabiting pathogens. Unfortunately, chemical fungicides often fail to effectively contact and eliminate vascular-inhabiting pathogens, because of their unique ecological niche in plants. Hypovirulence-associated mycoviruses can cause debilitation of fungal growth and virulence; but their fungal hosts are still capable of penetrating plant cells, as observed in vascular pathogens such as *Fusarium oxysporum* [54–56], and as we observed with the VdOMV2-infected Vd1–25 that had enhanced cellophane penetration ability. Some mycoviruses can even convert phytopathogenic fungi into beneficial endophytic fungi that can basic plant immune responses [3,57,58]. These biological features of hypovirulence-associated mycoviruses provide two potential strategies for controlling vascular fungal diseases. Mycovirus-mediated hypovirulent strains can serve as donor strains for transmitting mycoviruses to plant pathogen populations, thereby reducing their virulence. Additionally, mycoviruses can induce plant resistance to inhibit pathogen growth within plant cells. In this study, VdOMV2 infection enhanced the penetration ability and demonstrated effectiveness in the biocontrol of Verticillium wilt of cotton, which suggests that VdOMV2 has the potential to improve the colonization ability of hypovirulent strains (such as Vd1–25), thereby converting a virulent strain into a hypovirulent strain via VdOMV2 transmission within the plant vascular bundle. Therefore, VdOMV2 could be related to hypovirulence, and may be a promising candidate for vascular disease control. Infection by multiple mycovirus species has been observed in another vascular phytopathogenic fungus, *Fusarium oxysporum*, some of which have been related to hypovirulence. For instance, Fusarium oxysporum partitivirus 1 and Fusarium oxysporum ourmia-like virus 1 have shown promise as biocontrol agents against Fusarium wilt in bitter gourd [59,60]. Additionally, Fusarium oxysporum f. sp. dianthi virus 1 (FodV1) [54] induces hypovirulence, and regulates the colonization dynamics and spatial distribution of *F. oxysporum* in the xylem vessels of carnation plants (*Dianthus caryophillus*), resulting in delayed and restricted colonization by this pathogenic fungus [55,56]. Therefore, the discovery of additional hypovirulence-associated mycoviruses in a vascular-inhabiting phytopathogenic fungus could provide potential opportunities for biocontrol of vascular diseases. However, the application of mycovirus-mediated hypovirulent strains in managing these diseases will face challenges, including determining the precise timing for stable colonization in plant vascular systems and overcoming vegetative incompatibility barriers among fungi. Further research remains to address these questions.

VdOMV2 regulates the conversion from hyphal to microsclerotial colony types in *V. dahliae,* albeit partially. Melanin biosynthesis and microsclerotial production are crucial for *V. dahliae* in adapting to unfavorable environments, with melanin providing resistance to UV irradiation and high temperature [9,10], while microsclerotia confer resilience to adverse conditions, ultimately enabling prolonged survival within the soil [61]. VdOMV2 enhances melanin and microsclerotial biosynthesis by regulating melanin pathway genes, boosting abiotic stress tolerance in mycovirus-infected *V. dahliae*. Interestingly, VdOMV2 can only persist when microsclerotia-related genes are intact; when these genes are knocked out, VdOMV2 fails to maintain stable existence (Fig 5F). This unique dependency on microsclerotia-related genes highlights the critical role of microsclerotia in the survival and stability of VdOMV2, where infected isolates can colonize and dominate key agroecosystem niches, to enhance persistence and competitive fitness. Hypovirulence-associated mycoviruses generally reduce pigmentation and virulence of phytopathogenic fungi [62], as exemplified by Stemphylium lycopersici alternavirus 1, which negatively regulates polyketide synthase 1 to inhibit Altersolanol A synthesis in *Stemphylium lycopersici*, resulting in hypovirulence and melanin defects [63]. Deletion of several polyketide synthases also results in a significant reduction in the virulence of *V. dahliae* [8,30,64], suggesting that melanin biosynthesis is positively correlated to virulence in *V. dahliae*. Conversely, VdOMV2 positively regulates melanin and microsclerotial biosynthesis by regulating genes involved in melanin biosynthesis, but is associated with negative modulation of virulence in *V. dahliae*. Currently, this postulated phenomenon, with concurrent reduction in fungal host pathogenicity and enhancement of fungal host survival, has not been documented for other mycoviruses. VdOMV2 provides a system for exploring the correlation between melanin and virulence of phytopathogenic fungi.

In summary, VdOMV1 and VdOMV2, two *V. dahliae-*infecting ormycoviruses, were molecularly characterized. VdOMV2 could regulate melanin biosynthesis and microsclerotial formation, mediating the conversion from the hyphal to microsclerotial colony types in *V. dahliae*. Importantly, VdOMV2 was associated with significantly attenuated *V. dahliae* virulence, while potentially enhancing its penetration ability and resistance to adverse abiotic stress conditions. Therefore, the practical use of strains with VdOMV2-mediated hypovirulence, may offer a promising biocontrol strategy for managing Verticillium wilt of cotton, as well as setting an example that may be applicable for the control of other vascular fungal diseases.

## Materials and methods

### Fungal strains and culture conditions

Thirty strains of *V. dahliae* were obtained from the Laboratory of Microbial Resources and Utilization at Tarim University, Xinjiang (sequencing library named VdA1). A total of 228 strains were newly isolated from diseased cotton stalks in Xinjiang (VdA2-VdA7). A total of 123 strains were obtained from the National Key Laboratory of Crop Genetic Improvement at Huazhong Agricultural University (VdA8-VdA9). The strain Vd08284 was infected by VdPV1 [13]. All strains were cultured on PDA at 25°C and stored in 20% glycerol at -80°C. Microsclerotia were observed by aliquoting conidial suspensions of *V. dahliae* strains onto cellophane-covered PDA, and incubating for 12–15 days.

### RNA extraction, metatranscriptomic sequencing, and bioinformatic analyses

Total RNA was extracted using a TRIzol RNA extraction kit (TaKaRa, Dalian, China) and treated with DNase I. The cDNA libraries were then constructed and analyzed using an Illumina HiSeq 2500 platform (San Diego, CA, USA) at Shanghai Biotechnology Corporation. Adapter sequences and low-quality reads were removed using the Trimmomatic program (v0.32) [65]. The reads were aligned to the genome of *V. dahliae* strain VdLs.17 (GenBank assembly accession: GCA_000150675.1, ASM15067v2) with HISAT2 (v2.1.0) [66] and SAMtools (v1.9) [67]. Unmapped reads were extracted, and *de novo* assembly was performed with Trinity (v2.5.0) [68], yielding assembled alleles. These alleles were analyzed against a non-redundant protein database using the DIAMOND software [69].

### Detection of the mycovirus in *V. dahliae* strains

Total RNA was extracted and purified from individual strains. It was then reverse-transcribed into cDNA using M-MLV (Takara, Dalian, China). Specific primers, listed in S2 Table and designed based on mycoviral contig sequences, were used for RT-PCR to detect putative mycoviruses in the individual strains.

### Determination of the mycoviral terminal sequences

To further characterize the mycoviruses represented by the distinct contigs identified from RNA sequencing, we designed specific primers using Primer Premier 5 (Premier Biosoft Interpairs, Palo Alto, CA) for RT-PCR and RACE-PCR (S2 Table) to obtain the full genomic sequences of the mycoviruses. The terminal sequences of the genomic RNAs were determined using the SMARTer RACE 5′/3′ kit (Clontech Laboratories, California, USA), following the manufacturer’s instructions. For the 5′ terminus, an additional RNA template was synthesized by appending a known adapter sequence to the 5′ end of the RNA, which was then used in nested PCR. For the 3′ terminus, a polyadenylated tail was added to the RNA strand prior to cDNA synthesis. Nested PCR was performed with a universal primer mixture (S2 Table) to identify the terminal sequences of VdOMV1 and VdOMV2. PCR products were then purified, cloned, and sequenced. Additionally, the complete cDNA of the mycovirus was amplified using specific primers (S2 Table) and re-sequenced to confirm the genomic sequence.

### Multiple alignment and phylogenetic analysis

To elucidate the phylogenetic relationships of mycoviruses, RdRp sequences were aligned using MAFFT, and unreliable regions were trimmed with trimAl (v1.4) [70]. A maximum likelihood phylogenetic tree was constructed using IQ-TREE (v1.6.11) [71], employing the optimal amino acid substitution model identified by ModelFinder [72]. The resulting tree was visualized using FigTree (v1.44) (https://github.com/rambaut/figtree/releases). Additionally, a percentage consistency matrix between protein sequences was generated using Clustal Omega [73], and aligned sequences were visualized with Jalview (v2.11.0) [74]. The trees included established ormycoviruses of multiple alignment and phylogenetic analysis listed in S5 Table, and representative sequences from Supergroup024, Supergroup026 and Qin-Yue lineages published by Hou et al [20]. (The database available at https://db.cngb.org/search/project/CNP0005901/.)

### Virion extraction and purification

Mycovirus-infected strains were grown on cellophane-covered PDA plates for 5 days. The mycelia were collected, mixed, and disrupted in 0.1 M sodium phosphate buffer. Purified virions were obtained by CsCl gradient ultracentrifugation following a previously reported method [75]. The purified virions were negatively stained with Pure Terephthalic Acid and their morphology was observed using transmission electron microscopy.

### Western blotting and LC-MS/MS

SDS-PAGE gels (10%) were used to detect different gradients of purified virions and total proteins extracted from *V. dahliae* strains. Proteins were transferred to PVDF membranes and blocked with a Tris-buffered saline solution containing skimmed milk powder and Tween20. After incubating overnight at 4°C with a polyclonal antibody against the RNA2-encoded proteins of VdOMV1 and VdOMV2, the membranes were washed and incubated with a goat anti-rabbit secondary antibody. Enhanced chemiluminescence was used for detection. Another set of SDS-PAGE gels were used for different gradients of purified virions and stained with Thomas Brilliant Blue R250. Quantitative analysis of the data was performed using ImageJ software. Individual protein bands were extracted and analyzed using LC-MS/MS by Wuhan Jichuang Bioengineering, China.

### RNase A assay

The RNase A treatment method, adapted from a previously reported protocol [75], was used to assess whether VdOMV1 and VdOMV2 are capsidless viruses. Mycelial homogenates from *V. dahliae* strains Vd1–6, Vd1–25, and Vd080 were collected and treated with RNase A at varying concentrations and incubation times to degrade exposed ssRNA and dsRNA. Specifically, RNase A was applied at concentrations of 10 µg/mL for 30 min, 50 µg/mL for 1 h, or 100 µg/mL for 2 h. The capsid-containing virus VdPV1 served as a positive control, while the capsidless magoulivirus VdMoV1 was used as a negative control. Following RNase A treatment, RNA was extracted using phenol extraction, followed by PCIA (Phenol-Chloroform-Isoamyl Alcohol) and CIA (Chloroform-Isoamyl Alcohol) purification, and precipitated with LiCl. cDNAs were synthesized using M-MLV reverse transcriptase with random hexadeoxyribonucleotide primers (Takara, Dalian, China) and verified by PCR amplification with virus-specific primers listed in S2 Table.

### Pathogenicity assay

The strains of *V. dahliae* were inoculated into 100 mL of liquid CM medium and incubated at 28°C with shaking at 150 rpm for 4 dpi. The conidial suspension of each strain was obtained by filtration, harvested by centrifugation, washed 3 times, and then dissolve in sterile ddH_2_O. Conidia were harmonized to a concentration of 1 × 10^8^ conidia/mL using a hemocytometer. Two healthy cotton seedlings were placed in each pot, and at the one-real leaf stage, 20 mL of conidial suspension were added to each pot. Ten replicate pots were prepared for each strain. Plant conditions were assessed at 45 dpi using a disease rating scale, with level 0 indicating healthy seedlings and levels 1–4 indicating increasing symptom severity.

The experiments for biocontrol potential included an inoculation method similar to that previously described, but with two inoculations. In the first inoculation, 10 mL of a conidial suspension (1 × 10^8^ conidia/mL) from strains Vd1–25, Vd1–25VF-V2, and Vd1–25VF were applied to pots at the one-true leaf stage. Five days later, a second inoculation followed with 10 mL of conidial suspension from of strain Vd1–25VF, as shown in S7 Fig. Each group consisted of 10 replicates.

### Transcriptome analysis

Strains Vd1–25 and Vd1–25 VF were cultured on cellophane overlying PDA at 25°C for 15 days, with three biological replicates for each group. Total RNA was extracted using TRIzol, and sequencing was performed on the Illumina NovaSeq 4000 platform, generating 150-bp paired-end reads totaling over 6 GB of data. Clean reads were aligned to the *V. dahliae* reference genome with HISAT2 (v2.1.0), and transcripts were assembled using Stringtie (v1.3.4) [76]. DEGs were identified using pairwise comparisons with DESeq2 [77].

To validate the transcriptomic data, total RNA was extracted, reverse transcribed, and subjected to qRT-PCR with specific primers (S3 Table). The qRT-PCR was performed using TransStart Tip Green qPCR SuperMix (Transgen, Beijing, China). The amplification procedure included pre-denaturation at 94°C for 30 seconds, denaturation at 94°C for 5 seconds, and extension at 58°C for 30 seconds (40 cycles). Three independent biological and technical repeats were performed.

### Determination of fungal biomass

DNA extraction from cotton roots, leaves, and stalks was performed using the 4% CTAB method. qPCR amplification, with *V. dahliae*-specific primers for actin and cotton-specific primers for UBQ7 as controls (S3 Table), was conducted to detect fungal biomass in cotton stalks, roots, and leaves under different treatments. This experiment was repeated using at least three biological repeats, with each treatment set having three replicates.

### Generation of gene deletion mutants

Gene deletion vectors were constructed meticulously to ensure a systematic approach (S9 Fig). First, the flanking regions (ranging from 0.8 to 1.5 kb) of the target genes were amplified from strain Vd1–25 genomic DNA using specific primer pairs that included adapter sequences. Thereafter, the 2.1-kb hygromycin phosphotransferase (*hph*) gene sourced from vector pUCH18 was utilized as a template for cloning both the forward sequences of *hph* (HP) and the reverse sequences of *hph* (PH). The amplified fragments were ligated to both ends of *hph* through homologous recombination utilizing the ClonExpress II One Step Cloning Kit (Vazyme, Nanjing, China). The resulting recombinant plasmids were subsequently introduced into competent *Escherichia coli* DH5α cells and screened for kanamycin resistance to isolate positive clones. Concurrently, the two overlapping fragments were transferred into protoplasts of strain Vd1–25. Transformants resistant to hygromycin were selected on regeneration agar medium containing 100 μg/mL hygromycin B. Finally, the putative deletions were verified through diagnostic PCR using internal specific primer pairs of target and resistance hygromycin phosphotransferase (*hph*) genes were carried out, and primers are listed in S4 Table.

### Extraction and purification of melanin

*V. dahliae* strains were cultured in PDB at 28°C in darkness for 10 days, and mycelia were harvested and dried overnight at 50°C. Equal amounts of dried mycelia from each strain were ground into powder in liquid nitrogen and placed in 50 mL centrifuge tubes. Then, 10 mL of distilled water was added, and the mixture was boiled for 5 min and centrifuged at 12000 rpm for 5 min. The samples were washed with ddH_2_O and centrifuged before being dissolved in 10 mL NaOH solution (1 mol/L). The solution was then precipitated with 10 mL HCl (7 mol/L). After centrifugation at 10000 rpm for 15 min, the precipitate was dissolved in 10 mL NaOH solution, re-precipitated with 10 mL HCl, and centrifuged again at 10000 rpm for 15 min. The precipitate was washed three times with ddH_2_O, dried overnight at room temperature, and the dried material obtained was considered to contain mostly intracellular melanin. Then, the dried intracellular melanin was dissolved in 5 mL NaOH solution for 2 hours. After centrifugation at 10000 rpm for 10 min, the supernatant was transferred to a new centrifuge tube, and the absorbance was measured at 400 nm to assess the melanin content.

### Assessment of VdOMV2-infected strain sensitivity to abiotic stress

To assess the responses of the VdOMV2-infected strain Vd1–25, virus-free strain Vd1–25VF, and VdOMV2-infected strain Vd1–25VF-V2 to various stress conditions, their growth rates were measured on PDA supplemented with 0.7 M, 1 M, and 1.5 M sorbitol, as well as 0.5 M, 0.7 M, and 1 M NaCl and KCl [8,40]. Colony diameters were recorded every two days from the fourth to the fourteenth day of incubation, and the relative growth inhibition rate was calculated as [(control colony diameter − treatment colony diameter)/control colony diameter] × 100%.

For the thermostability assay, conidial suspensions (1 × 10⁵ conidia/mL) of all three strains were incubated at 40°C, 45°C, and 50°C for 10 min, while a control group was maintained at 25°C [78]. To evaluate UV sensitivity, conidial suspensions were placed in disposable plastic Petri dishes and the lids opened to expose cultures to UV irradiation at 0 mJ, 25 mJ, 50 mJ, and 100 mJ in a UV crosslinker (HL-2000 Hybrilinker, UVP, Upland, CA, USA). The treated conidia were then spread onto PDA plates and incubated for 3 and 6 days before assessment. The relative conidial germination rate was determined as [(control conidial germination number − treatment conidial germination number)/control conidial germination number] × 100%.

### Mycelial penetration assays

To evaluate the penetration ability of strains Vd1–25 and Vd1–25VF, both strains were cultured on cellophane overlying PDA for 3 days. After removing the cellophane, the cultures were incubated for an additional 3 days to observe whether there was subsequent hyphal indicating penetration.

## Supporting information

S1 Fig RT-PCR confirmation of *de novo* assembled contigs from strains Vd1–6 and Vd1–25 generated by Illumina sequencing. (TIF)

S2 Fig The maximum-likelihood phylogenetic tree was constructed using representative sequences from Supergroup024 and established ormycoviruses. Supergroup026 and Qin-Yue lineages served as outgroups, based on prior taxonomic classification by Hou et al. The ormycoviruses are divided into five major clades (Group 1 to Group 5), with VdOMV1 and VdOMV2 highlighted in red font. The scale bar (tree scale = 1) indicates the number of substitutions per site.
(PDF)

S3 Fig VdOMV1 and VdOMV2 are capsidless viruses. *(A)* Co-culture of the VdPV1-infected strain Vd08284 as donor and the VdOMV2-infected strain Vd1–25 as recipients for 15 dpi (upper panel) on PDA at 25°C. Colony morphology (lower panel) of Vd1–25 derivative isolates that were picked up from the position marked with red asterisks in the upper panel. *(B)* A simple procedure for the extraction of virion VdPV1 from strain Vd1–25-P, and the components in the extraction. Created in BioRender. Jiamin, G. (2025) https://BioRender.com/aaawfnb. *(C)* Transmission electron microscopy of the virus particles purified from the strain Vd1–25-P co-infected by VdOMV2 and VdPV1. The red arrow points to a virion of VdPV1, and the scale bar represents 200 nm. *(D)* Coomassie Brilliant Blue staining of VP proteins in the in SDS-PAGE gel. M refers to protein marker. The major protein band (approximate 50 kDa) enclosed in a red square was used for subsequent peptide mass fingerprinting (PMF) and N-terminal sequencing. *(E)* Result of PMF analysis of the major 50-kDa protein in a strain co-infected by VdOMV2 and VdPV1. Peptide sequences identified by PMF analysis were mapped to the amino acid of the protein encoded by VdPV1 ORF2 as denoted by the red letters. The yellow triangle indicates the cleavage site determined by N-terminal sequencing of the 50-kDa protein band.
(TIF)

S4 Fig VdOMV2 levels in strains Vd1–25 and Vd1–25-P were detected by qRT-PCR. The data were analyzed by Student’s t-test. Statistical significance is indicated as follows ***0.0001 < *P* < 0.001; and error bars represent standard deviation (SD).
(TIF)

S5 Fig VdOMV1 and VdOMV2 affect the biological characteristics of *V. dahliae.* *(A)* Western blot detection of ormycoviruses with antibody for RNA2-encoded proteins of VdOMV1 and VdOMV2. *(B)* The colony morphology of strains infected by ormycoviruses was observed on PDA (25°C, 15 dpi). *(C)* and *(D)* Pathogenicity assay of strains infected by ormycoviruses on live cotton seedlings inoculated with 10^8^/ml conidia, and Verticillium wilt symptom *(C, upper panel)* were photographed at 45 dpi on cotton along with vascular discoloration *(C, lower panel)*. Data were analyzed by one-way ANOVA, and error bars represent the SD, ns*P > *0.999; **0.001* < P < *0.01.
(TIF)

S6 Fig Horizontal transmission of VdOMV2. *(A)* Co-culture of the VdOMV2-infected strain Vd1–25 (donor strain) and VdOMV2-free strain (recipient strain) at 15 dpi *(upper panel)* on PDA at 25°C. Colony morphology *(lower panel)* of Vd1–25VF-derivative isolates that were picked up from the position marked with red asterisks in the upper panel. *(B)* The horizontal transmission efficacy of VdOMV2 on PDA using RT-PCR with the specific-primers for VdOMV2.
(TIF)

S7 Fig The detailed protocol for evaluating the biocontrol potential of the VdOMV2-infected strain. Created in BioRender. Jiamin, G. (2025) https://BioRender.com/c8git0b.
(TIF)

S8 Fig Correlation analysis of gene expression in RNA-Seq samples. *(A)* Heatmap of the squared value of the Pearson correlation coefficient between the sequenced samples. The squared value within and between groups is based on the read count value of all genes in each sample. The higher the correlation coefficient between samples, the more similar their gene expression patterns. *(B)* Principal component analysis (PCA) analysis was performed on the read counts of all sample genes. The x and y axes respectively represent the first and second principal components. The value in the brackets of the axis label represents the percentage of the overall variance explained by the principal components.
(TIF)

S9 Fig Gene deletion construction based on the split-marker approach. *(A)* and *(B) VdSCD1* and *VdBrn1* gene deletion construct containing the hygromycin phosphotransferase (*hph*) cassette. *(C)* and *(D)* Identification of the knockout mutant of *VdSCD1* and *VdBrn1* by PCR. “U” represents the upstream flanking sequence of the gene, “D” represents the downstream flanking sequence of the gene, “H” represents the hygromycin B phosphotransferase gene, and “G” represents the *VdSCD1* and *VdBrn1*. The associated primer sequences were used for detection. The expected sizes of the PCR product and the primer sequences are listed in S4 Table.
(TIF)

S10 Fig Melanin production was assessed in ΔVdSCD1 and ΔVdBrn1 deletion mutants. *(A)* Melanin from culture filtrates of the wild type (WT) strain Vd1–25 and the *ΔVdSCD1* and *ΔVdBrn1* deletion mutants. *(B)* The amount of melanin in WT and deletion mutants, asterisks represent significant differences (data were analyzed using one-way ANOVA, ****P < 0.0001).
(TIF)

S1 Table Assembled sequences with similarity to previously described viruses. (DOCX)

S2 Table Primers used for PCR analysis in this study. (DOCX)

S3 Table Primers used for qRT-PCR analysis in this study. (DOCX)

S4 Table Primers used for vector construction in this study. (DOCX)

S5 Table The list of viruses used in alignment analysis and phylogenetic analysis in this study. (DOCX)
