# Supplementary figures and images for: A hypovirulence-associated capsidless bi-segmented ssRNA mycovirus enhances melanin and microsclerotial production in a vascular phytopathogenic fungus
Source: PLoS Pathog. 2025 Aug 11;21(8):e1013348. doi: 10.1371/journal.ppat.1013348 (PMC12360652; doi:10.1371/journal.ppat.1013348)

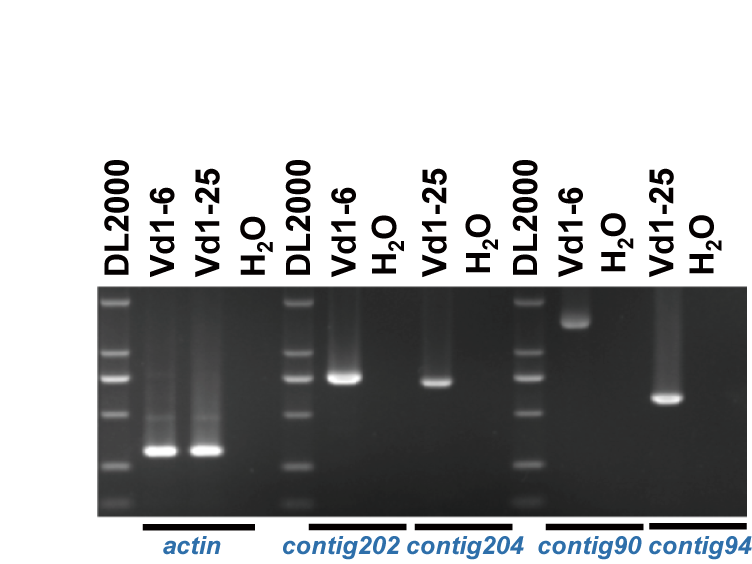

Supplement: S1 Fig — (TIF) [file ppat.1013348.s001.tif]

Tree scale: 1

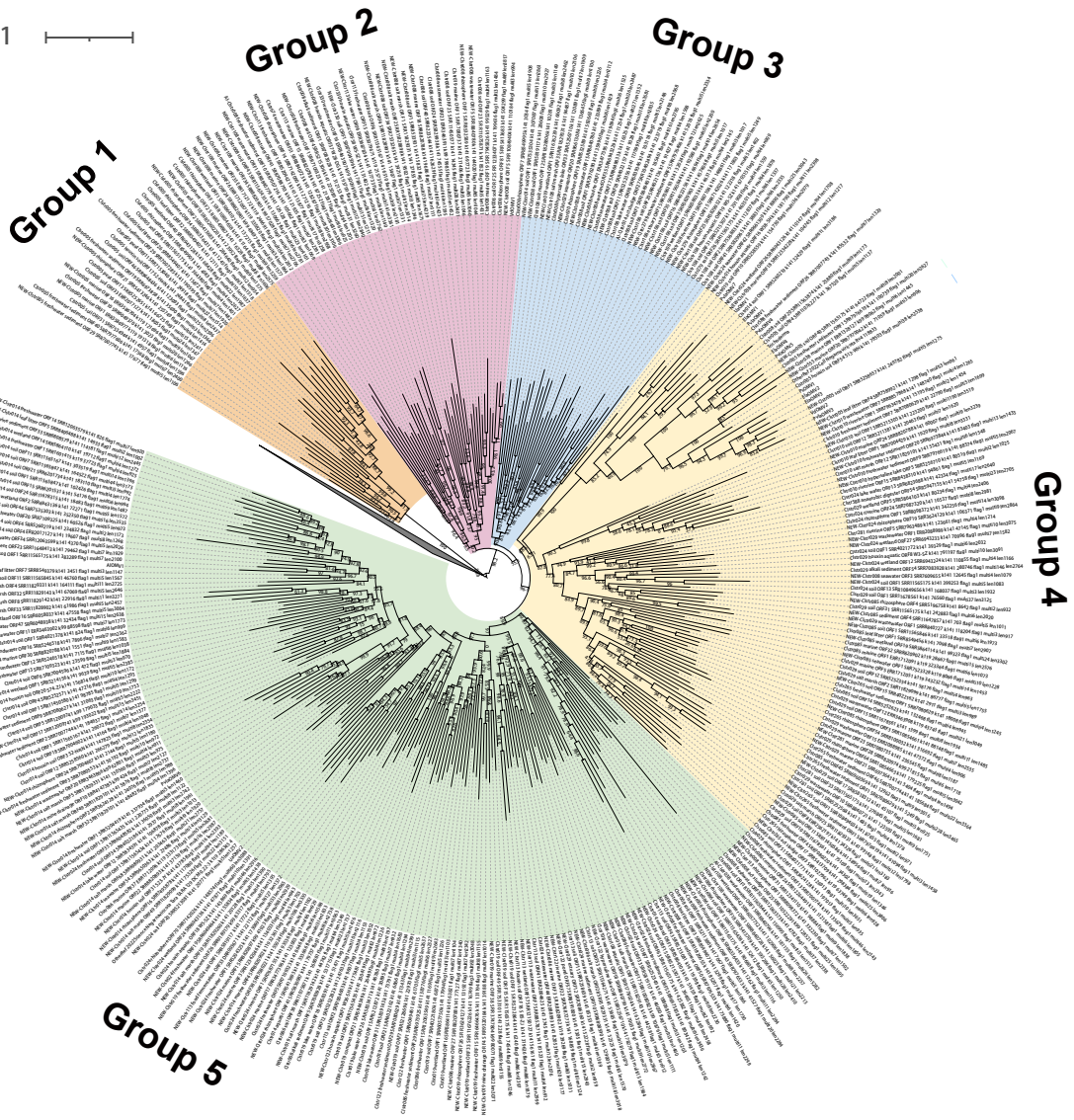

Supplement: S2 Fig — Supergroup026 and Qin-Yue lineages served as outgroups, based on prior taxonomic classification by Hou et al. The ormycoviruses are divided into five major clades (Group 1 to Group 5), with VdOMV1 and VdOMV2 highlighted in red font. The scale bar (tree scale = 1) indicates the number of substitutions per site. (PDF) [file ppat.1013348.s002.pdf]

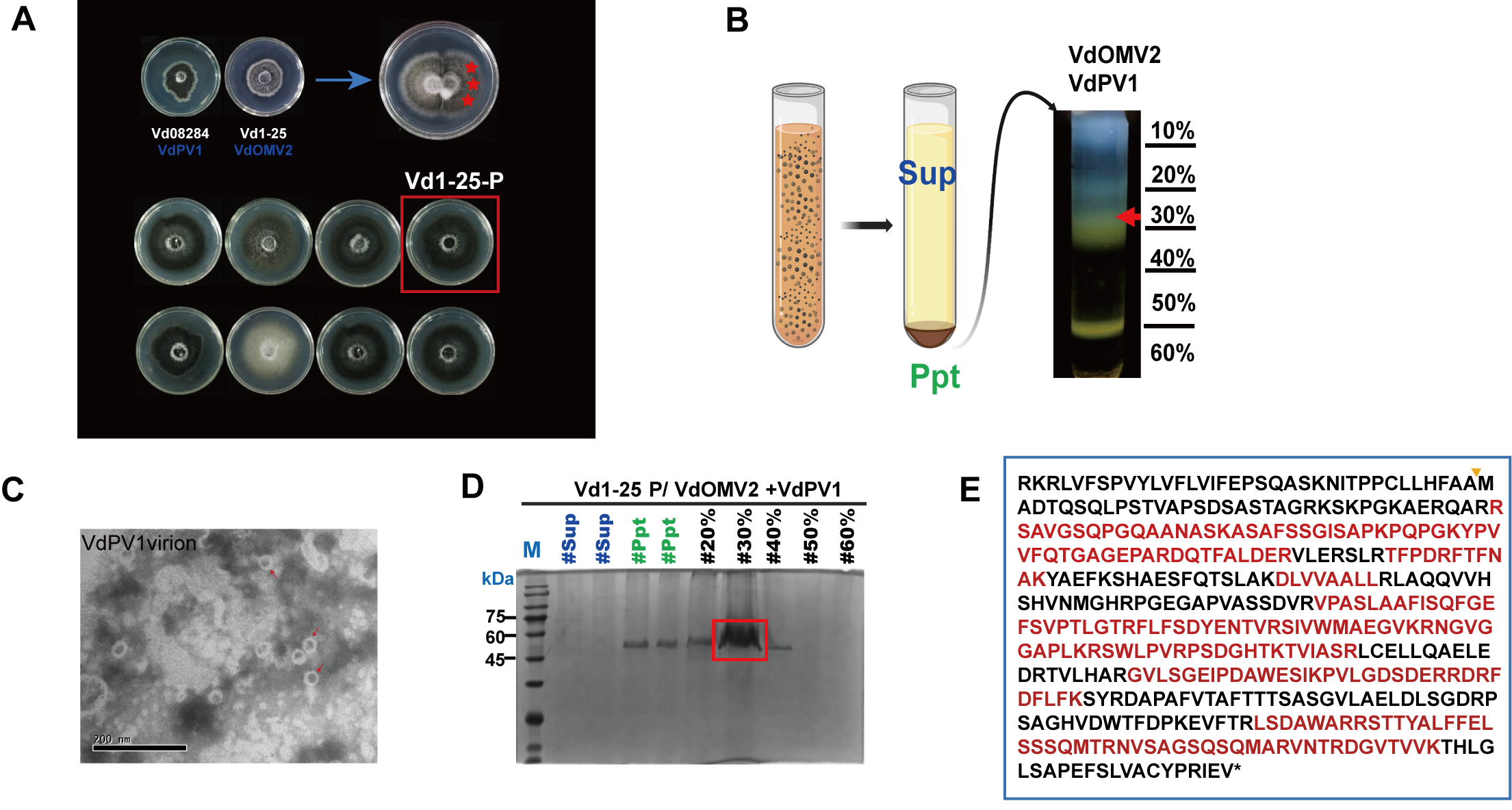

Supplement: S3 Fig — (A) Co-culture of the VdPV1-infected strain Vd08284 as donor and the VdOMV2-infected strain Vd1–25 as recipients for 15 dpi (upper panel) on PDA at 25°C. Colony morphology (lower panel) of Vd1–25 derivative isolates that were picked up from the position marked with red asterisks in the upper panel. (B) A simple procedure for the extraction of virion VdPV1 from strain Vd1–25-P, and the components in the extraction. Created in BioRender. Jiamin, G. (2025) https://BioRender.com/aaawfnb. (C) Transmission electron microscopy of the virus particles purified from the strain Vd1–25-P co-infected by VdOMV2 and VdPV1. The red arrow points to a virion of VdPV1, and the scale bar represents 200 nm. (D) Coomassie Brilliant Blue staining of VP proteins in the in SDS-PAGE gel. M refers to protein marker. The major protein band (approximate 50 kDa) enclosed in a red square was used for subsequent peptide mass fingerprinting (PMF) and N-terminal sequencing. (E) Result of PMF analysis of the major 50-kDa protein in a strain co-infected by VdOMV2 and VdPV1. Peptide sequences identified by PMF analysis were mapped to the amino acid of the protein encoded by VdPV1 ORF2 as denoted by the red letters. The yellow triangle indicates the cleavage site determined by N-terminal sequencing of the 50-kDa protein band. (TIF) [file ppat.1013348.s003.tif]

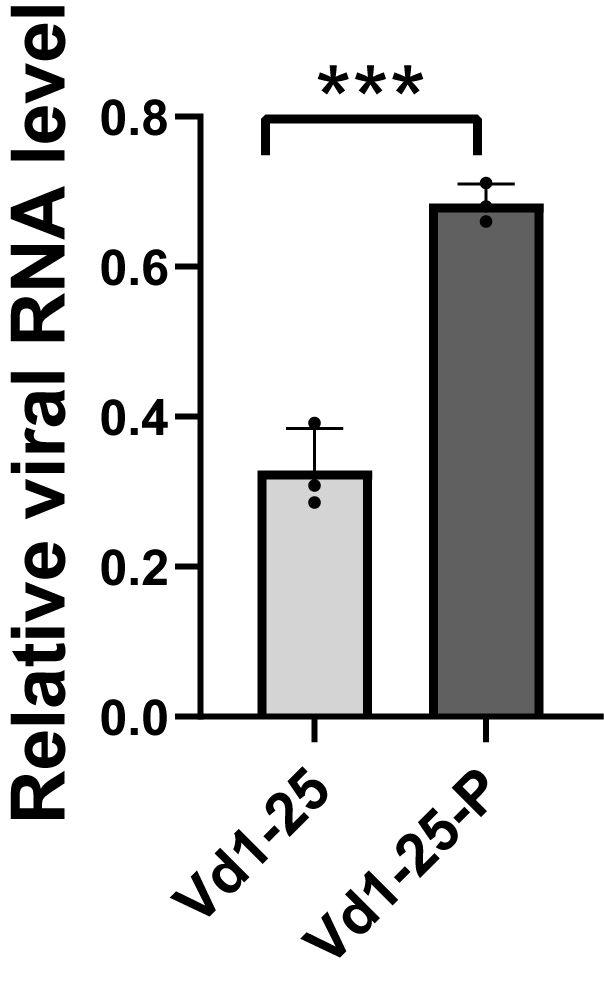

Supplement: S4 Fig — The data were analyzed by Student’s t-test. Statistical significance is indicated as follows ***0.0001 < P < 0.001; and error bars represent standard deviation (SD). (TIF) [file ppat.1013348.s004.tif]

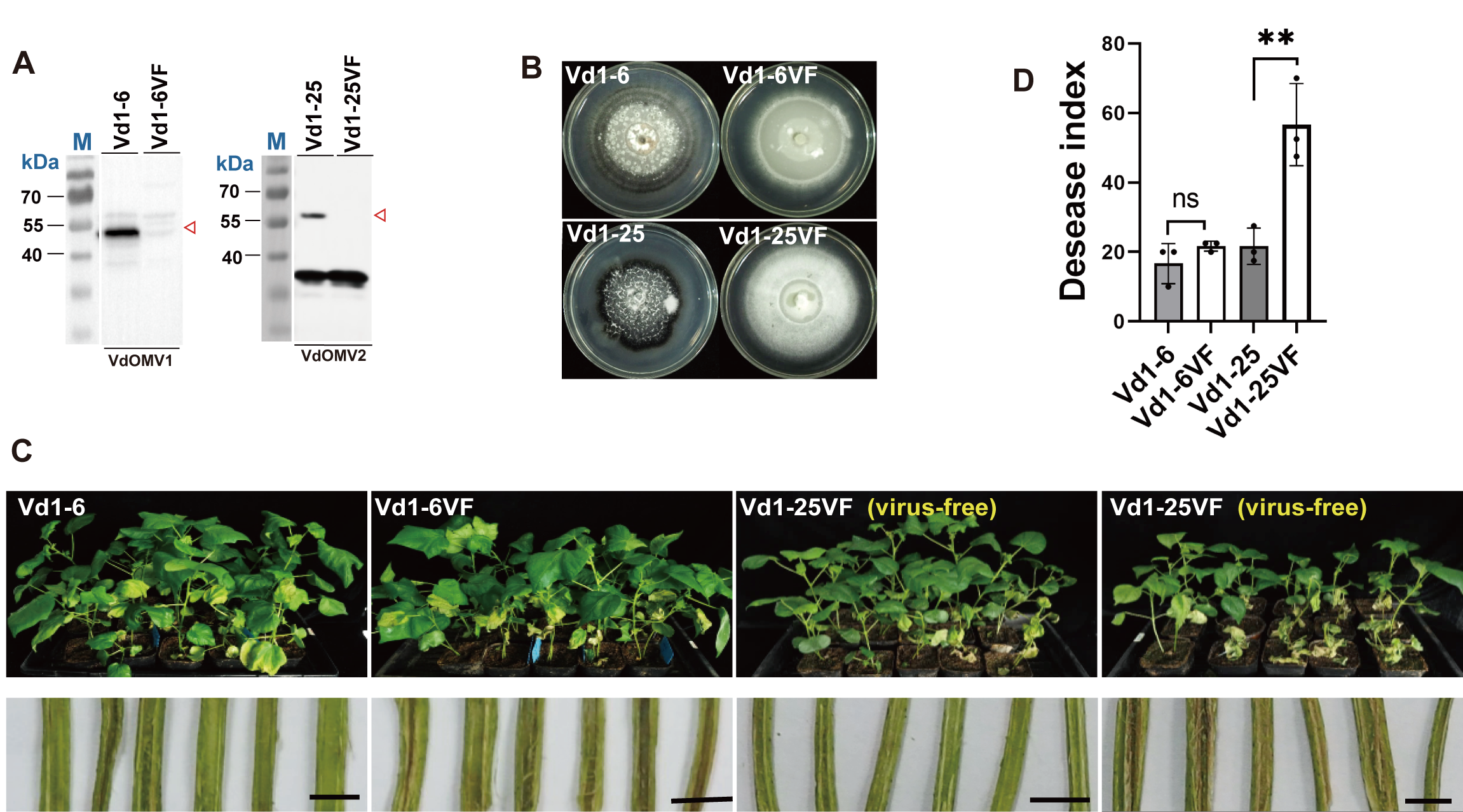

Supplement: S5 Fig — (A) Western blot detection of ormycoviruses with antibody for RNA2-encoded proteins of VdOMV1 and VdOMV2. (B) The colony morphology of strains infected by ormycoviruses was observed on PDA (25°C, 15 dpi). (C) and (D) Pathogenicity assay of strains infected by ormycoviruses on live cotton seedlings inoculated with 108/ml conidia, and Verticillium wilt symptom (C, upper panel) were photographed at 45 dpi on cotton along with vascular discoloration (C, lower panel). Data were analyzed by one-way ANOVA, and error bars represent the SD, nsP > 0.999; **0.001 < P < 0.01. (TIF) [file ppat.1013348.s005.tif]

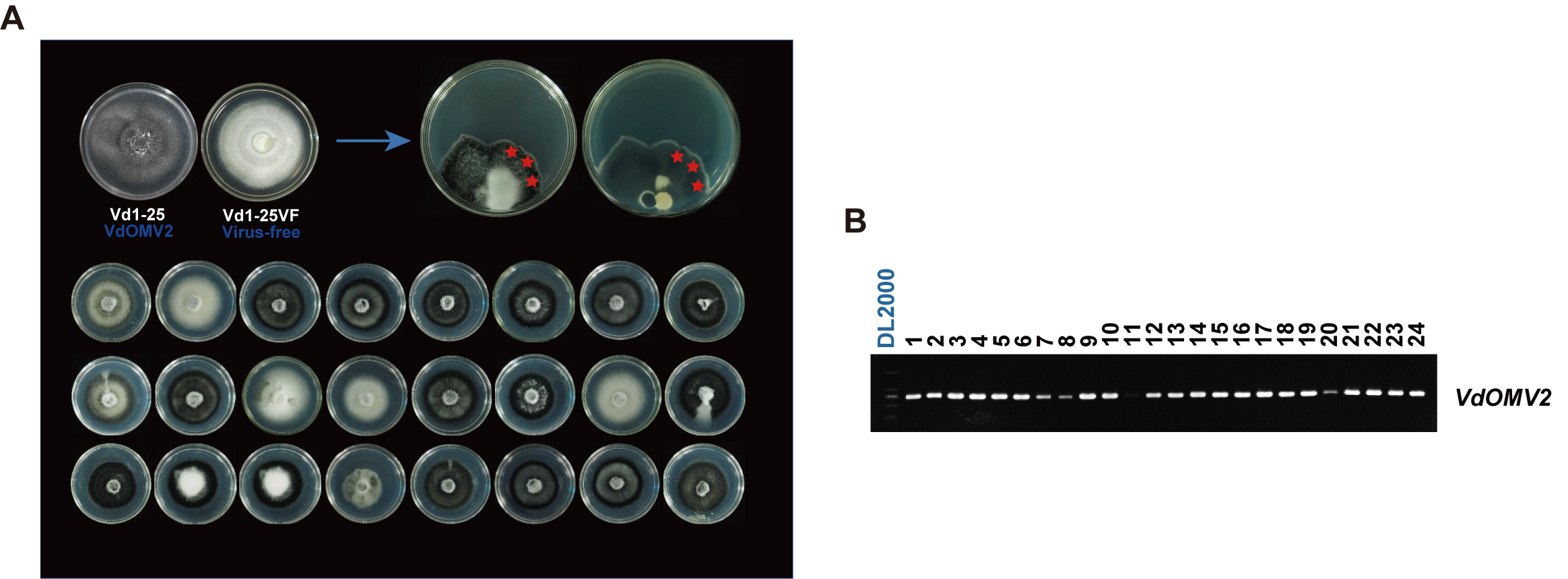

Supplement: S6 Fig — (A) Co-culture of the VdOMV2-infected strain Vd1–25 (donor strain) and VdOMV2-free strain (recipient strain) at 15 dpi (upper panel) on PDA at 25°C. Colony morphology (lower panel) of Vd1–25VF-derivative isolates that were picked up from the position marked with red asterisks in the upper panel. (B) The horizontal transmission efficacy of VdOMV2 on PDA using RT-PCR with the specific-primers for VdOMV2. (TIF) [file ppat.1013348.s006.tif]

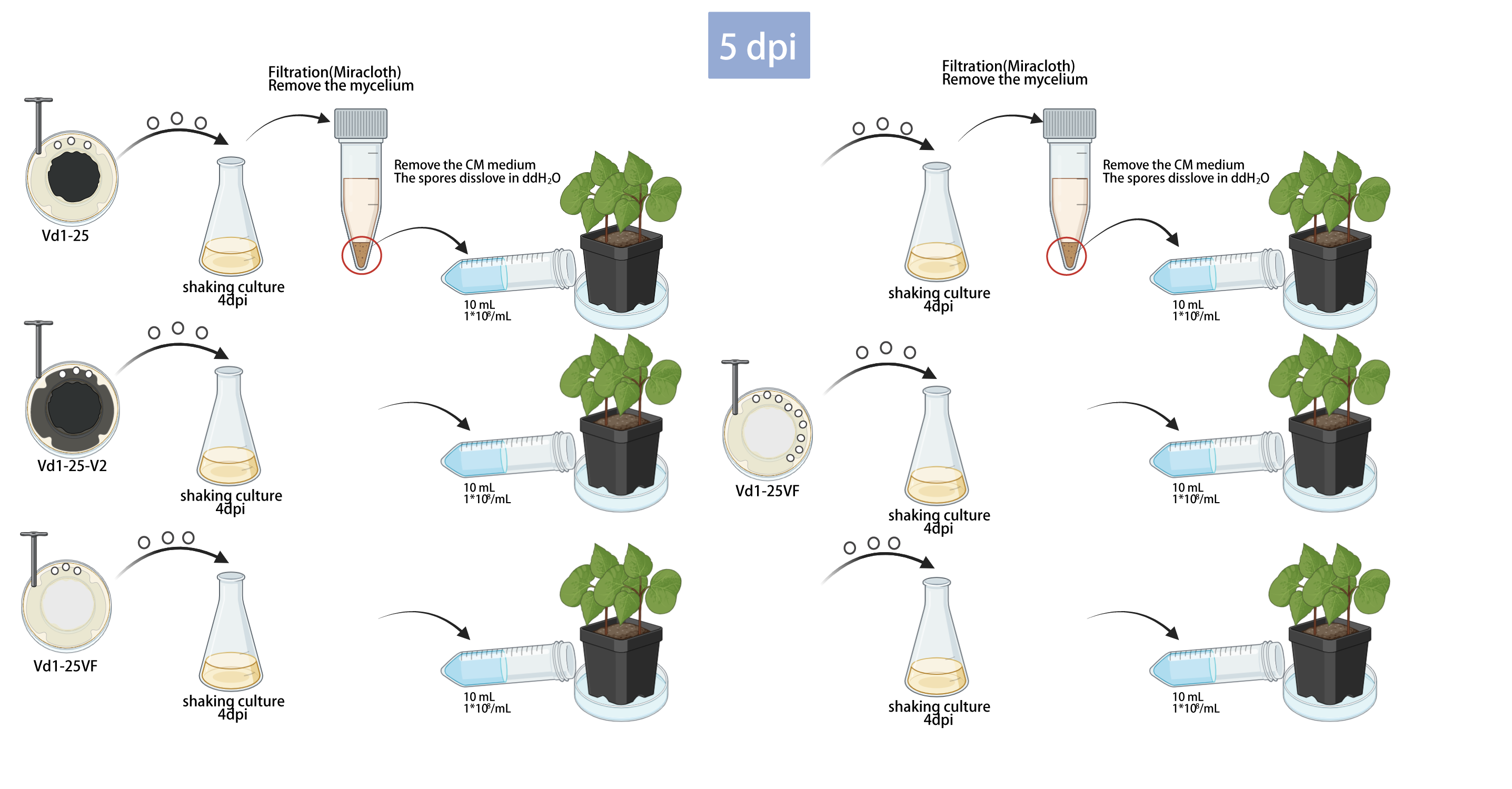

Supplement: S7 Fig — Created in BioRender. Jiamin, G. (2025) https://BioRender.com/c8git0b. (TIF) [file ppat.1013348.s007.tif]

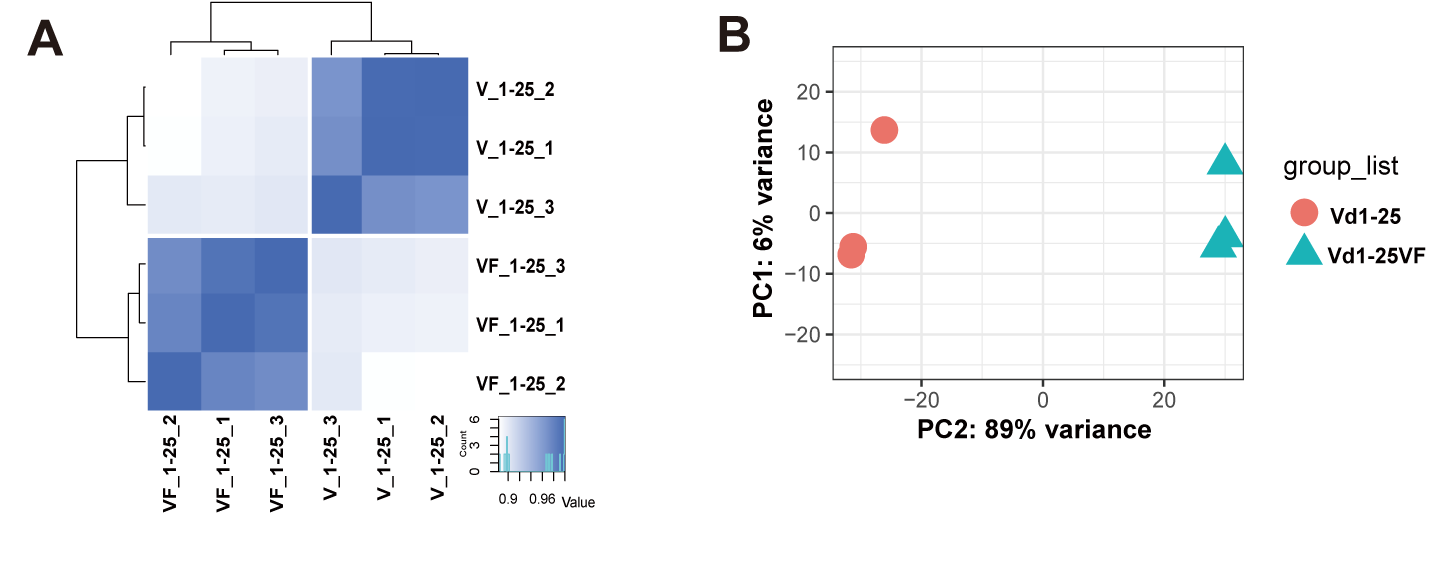

Supplement: S8 Fig — (A) Heatmap of the squared value of the Pearson correlation coefficient between the sequenced samples. The squared value within and between groups is based on the read count value of all genes in each sample. The higher the correlation coefficient between samples, the more similar their gene expression patterns. (B) Principal component analysis (PCA) analysis was performed on the read counts of all sample genes. The x and y axes respectively represent the first and second principal components. The value in the brackets of the axis label represents the percentage of the overall variance explained by the principal components. (TIF) [file ppat.1013348.s008.tif]

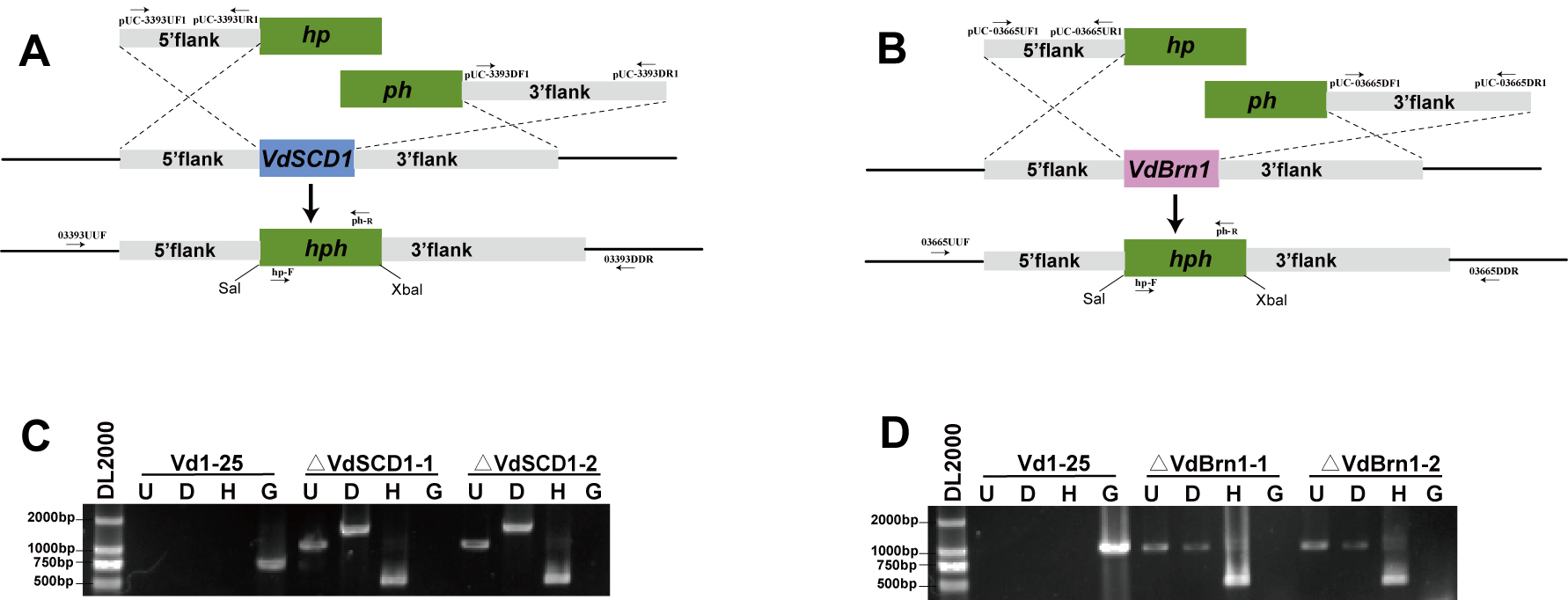

Supplement: S9 Fig — (A) and (B) VdSCD1 and VdBrn1 gene deletion construct containing the hygromycin phosphotransferase (hph) cassette. (C) and (D) Identification of the knockout mutant of VdSCD1 and VdBrn1 by PCR. “U” represents the upstream flanking sequence of the gene, “D” represents the downstream flanking sequence of the gene, “H” represents the hygromycin B phosphotransferase gene, and “G” represents the VdSCD1 and VdBrn1. The associated primer sequences were used for detection. The expected sizes of the PCR product and the primer sequences are listed in S4 Table. (TIF) [file ppat.1013348.s009.tif]

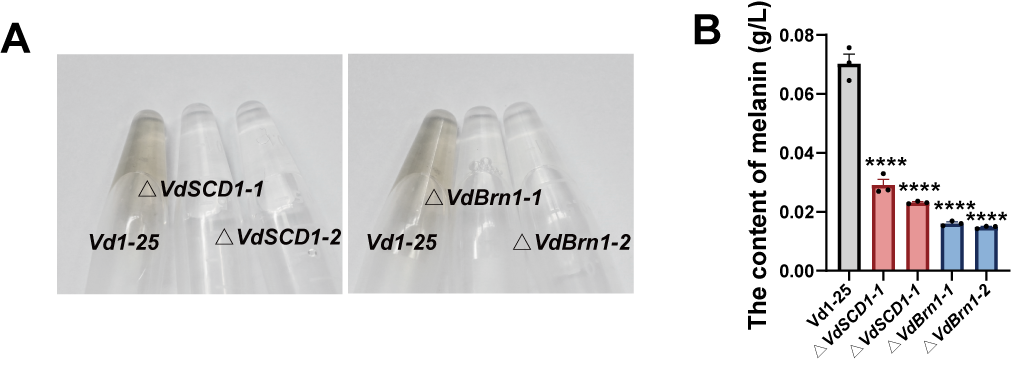

Supplement: S10 Fig — (A) Melanin from culture filtrates of the wild type (WT) strain Vd1–25 and the ΔVdSCD1 and ΔVdBrn1 deletion mutants. (B) The amount of melanin in WT and deletion mutants, asterisks represent significant differences (data were analyzed using one-way ANOVA, ****P < 0.0001). (TIF) [file ppat.1013348.s010.tif]
