# Supplementary material for: A hypovirulence-associated capsidless bi-segmented ssRNA mycovirus enhances melanin and microsclerotial production in a vascular phytopathogenic fungus
Source: PLoS Pathog. 2025 Aug 11;21(8):e1013348. doi: 10.1371/journal.ppat.1013348 (PMC12360652; doi:10.1371/journal.ppat.1013348)
Supplement: S1 Table — (DOCX) [file ppat.1013348.s011.docx]

## Table S1. Assembled sequences with similarity to previously described viruses.

| **librirary** | **Strains No** | **virus-**  **infected strains** | **contig name** | **contig length** | **virus name** | **Best hit** | **Aa identity** | **TPM** |  |  |  |
| --- | --- | --- | --- | --- | --- | --- | --- | --- | --- | --- | --- |
| VdA1 | 30 | Vd1-12  Vd1-6  Vd1-25  Vd1-27 | contig119 | 2975 | VdMoV1 | Verticillium dahliae magoulivirus 1 (UVD54632.1) | 99.67% | 1712.69 |  |  |  |
|  |  |  | contig193 | 2447 | VdOMV2 RdRp | Downy mildew lesion-associated ormycovirus 3 RdRp (USW07202.1) | 53.08% | 93.6452 |  |  |  |
|  |  |  | contig420 | 1710 | VdOMV2 HP | Downy mildew lesion-associated ormycovirus 3 HP (USW07203.1) | 50.21% | 154.793 | |  |  |
|  |  |  | contig80 | 3352 | VdOMV1 RdRp | Downy mildew lesion-associated ormycovirus 2 RdRp ([USW07197.1](https://www.ncbi.nlm.nih.gov/protein/USW07197.1?report=genbank&log$=prottop&blast_rank=2&RID=6ZSVVDTR016)) | 26.85% | 464.347 | | |  |
|  |  |  | contig539 | 1525 | VdOMV1 HP |  |  | 1219.11 | | | |
| VdA5 | 36 | Vd5-19 | contig9632 | 585 | VdOMV1 RdRp | Downy mildew lesion-associated ormycovirus 2 RdRp ([USW07197.1](https://www.ncbi.nlm.nih.gov/protein/USW07197.1?report=genbank&log$=prottop&blast_rank=2&RID=6ZSVVDTR016)) | 29.17% | 729.294 |  |  |  |
|  |  |  | contig5535 | 875 | VdOMV1 HP |  |  | 471.137 | |  |  |
|  |  |  | contig6580 | 773 | VdOMV2 RdRp | Downy mildew lesion-associated ormycovirus 3 RdRp (USW07202.1) | 45.06% | 118.977 |  |  |  |
|  |  |  | contig5243 | 904 | VdOMV2 HP | Downy mildew lesion-associated ormycovirus 3 HP (USW07203.1) | 49.69% | 156.142 | | | |
| VdA6 | 35 | Vd6-11  Vd6-13 | contig130 | 3129 | VdOMV1 RdRp | Downy mildew lesion-associated ormycovirus 2 RdRp ([USW07197.1](https://www.ncbi.nlm.nih.gov/protein/USW07197.1?report=genbank&log$=prottop&blast_rank=2&RID=7074BNN0013)) | 23.79% | 113.436019 |  |  |  |
|  |  |  | contig1078 | 1524 | VdOMV1 1 HP |  |  | 97.282617 |  |  |  |
|  |  |  | contig286 | 2437 | VdOMV 2 RdRp | Downy mildew lesion-associated ormycovirus 3 RdRp (USW07202.1) | 52.15% | 18.1375 | | | |
|  |  |  | contig855 | 1691 | VdOMV2 HP | Downy mildew lesion-associated ormycovirus 3 HP (USW07203.1) | 49.89% | 31.642 | | | |
| VdA9 | 62 | Vd9-17 | contig21928 | 625 | VdMoV1 | Verticillium dahliae magoulivirus 1  (UVD54632.1) |  | 956.15 |  |  |  |
| VdB1 | 2 | Vd1-6  Vd1-25 | contig 90 | 2479 | VdOMV1 RdRp | Downy mildew lesion-associated ormycovirus 2 RdRp (USW07197.1) | 26.85% | 1205.84 |  |  |  |
|  |  |  | contig94 | 2448 | VdOMV2 RdRp | Downy mildew lesion-associated ormycovirus 3 RdRp (USW07202.1) | 48.49% | 261.151 | |  |  |
|  |  |  | contig202 | 1717 | VdOMV1 HP |  |  | 2755.72 |  |  |  |
|  |  |  | contig204 | 1712 | VdOMV2 HP | Downy mildew lesion-associated ormycovirus 3 HP(USW07203.1) | 50.21% | 386.467 | | |  |
|  |  |  | contig110 | 2305 | VdMoV1 | Verticillium dahliae magoulivirus 1  (UVD54632.1) | 100% | 20048.3 | | | |
