# Supplementary material for: A hypovirulence-associated capsidless bi-segmented ssRNA mycovirus enhances melanin and microsclerotial production in a vascular phytopathogenic fungus
Source: PLoS Pathog. 2025 Aug 11;21(8):e1013348. doi: 10.1371/journal.ppat.1013348 (PMC12360652; doi:10.1371/journal.ppat.1013348)
Supplement: S2 Table — (DOCX) [file ppat.1013348.s012.docx]

## Table S2. Primers used for PCR analysis in this study.

| **Function** | | **Primer** | | **Sequence (5' to 3')** | | **Tm (℃)** | | **Product (bp)** | |
| --- | --- | --- | --- | --- | --- | --- | --- | --- | --- |
| Mycovirus primer | | VdOMV1-RNA1F | | TGCTGGGTTCGCTCTACTGTG | | 58 | | 747 | |
|  |  | VdOMV1-RNA1R | | GGCCAGGTTCAACCGGAATTG | |  |  |  |  |
|  |  | VdOMV1-RNA2F | | GGACAGAATGAAGGCGAGGTCT | | 58 | | 581 | |
|  |  | VdOMV1-RNA2R | | GCTCGTTCAAGCTCCGAATCTC | |  |  |  |  |
|  |  | VdOMV2-RNA1F | | ATGTGTAGCTCCTGGTGGCATT | | 58 | | 717 | |
|  |  | VdOMV2-RNA1R | | ATCGCTTGGGTGATCTTGGCT | |  |  |  |  |
|  |  | VdOMV2-RNA2F | | ACAACGCTTGCTGCTCTTTCTC | | 58 | | 575 | |
|  |  | VdOMV2-RNA2R | | AACGCTCCTAGCCGGAACTC | |  |  |  |  |
|  |  | VdMoV1-F | | ACGTTGGGCGCTGTAAAGGA | | 58 | | 693 | |
|  |  | VdMOV1 | | GACACGCAAGACGCACTGAC | |  |  |  |  |
| RACE-PCR | | RACE2 | | TCACTAAAGAATTCGATCGATC | |  | |  | |
|  |  | RACE3 | | CGATCGATCATGATGCAATGC | |  | |  | |
|  |  | VdOMV1-S1-5’-1 | | ACGAGCTTGAATCCGAAGTCCA | |  | |  | |
|  |  | VdOMV1-S1-5’-2 | | AAGCGTCCAGCCATTAGGTTC | |  | |  | |
|  |  | VdOMV1-S1-3’-1 | | TACGGAACCTGCTCTCACAGC | |  | |  | |
|  |  | VdOMV1-S1-3’-2 | | CCGAGGATTCTGATCCTGGCTT | |  | |  | |
|  |  | VdOMV1-S2-5’-1 | | GGACAGAATGAAGGCGAGGTCT | |  | |  | |
|  |  | VdOMV1-S2-5’-2 | | TCGGGAGATTAGCCTGGTGTTG | |  | |  | |
|  |  | VdOMV1-S2-3’-1 | | CAGGGCTGTCGCAAACAACC | |  | |  | |
|  |  | VdOMV1-S2-3’-2 | | AGCCATCGCCTTGGAATTTGTG | |  | |  | |
|  |  | VdOMV2-S1-5’-1 | | TTCTTCAGGAGGTGGCTTCTCA | |  | |  | |
|  |  | VdOMV2-S1-5’-2 | | TGCCCTTCTGGTAGGAGAGAGT | |  | |  | |
|  |  | VdOMV2-S1-3’-1 | | ACAATGGGCACATACCCGAAGA | |  | |  | |
|  |  | VdOMV2-S1-3’-2 | | TCCTGACGAGGATGGTCAAAGC | |  | |  | |
|  |  | VdOMV2-S2-5’-1 | | AAGCCTGGCCGGTAATATCACA | |  | |  | |
|  |  | VdOMV2-S2-5’-2 | | AGCGCGTTGACCAACCTCT | |  | |  | |
|  |  | VdOMV2-S2-3’-1 | | GTGGACCCAGTGTCCATCAAGA | |  | |  | |
|  |  | VdOMV2-S2-3’-1 | | GGCAAGATGCCTCTGGTACGA | |  | |  | |
