# Supplementary material for: A hypovirulence-associated capsidless bi-segmented ssRNA mycovirus enhances melanin and microsclerotial production in a vascular phytopathogenic fungus
Source: PLoS Pathog. 2025 Aug 11;21(8):e1013348. doi: 10.1371/journal.ppat.1013348 (PMC12360652; doi:10.1371/journal.ppat.1013348)
Supplement: S3 Table — (DOCX) [file ppat.1013348.s013.docx]

## Table S3. Primers used for qRT-PCR analysis in this study.

| **Function** | | **Primer** | **Sequence (5' to 3')** | | **Tm (℃)** | | **Product (bp)** |
| --- | --- | --- | --- | --- | --- | --- | --- |
| qRT-PCR | VdOMV2-QF1 | | AGCTGTGAAGTCTTTGGGACCA | | 58 | 182 |  |
|  | VdOMV2-QR1 | | GCCACCAGGAGCTACACATCTT | |  |  |  |
|  | Actin-QF1 | | ACTGCTCTTGCTCCTTCTTCCA | | 58 | 194 |  |
|  | Actin-QR1 | | ACCGCTCTCGTCGTACTCCT  GAATGTGGCGCCGGGACCTTC | |  |  |  |
|  | UBQ7F1 | |  |  | 58 | 101 |  |
|  | UBQ7R1 | | ACTCAATCCCCACCAGCCTTCTGG | |  |  |  |
|  | 04551-QF1 | | TCTCTGCGGTGGTGGTTATACC | | 58 | 135 |  |
|  | 04551-QR1 | | TCGGCGTAATCCTCAGCGTAG | |  |  |  |
|  | 06254-QF1 | | CGGTGGTGGTGCCTTCTCTT | | 58 | 145 |  |
|  | 06254-QR1 | | GCCGTTCGTGAATGTGAGAGC | |  |  |  |
|  | 04977-QF1 | | GGCACCAACATCACCTTCACTG | | 58 | 115 |  |
|  | 04977-QR1 | | TGCTGTTGTTGATGCGGATTGT | |  |  |  |
|  | 06165-QF1 | | TGCTTCTCTTCCCTCTTCGTCG | | 58 | 146 |  |
|  | 06165-QR1 | | CCGTCGGTCCACCATGAGTAA | |  |  |  |
|  | 00190-QF1 | | CTCGTCCTGATCCGTATCCCA | | 58 | 190 |  |
|  | 00190-QR1 | | TGTGAATTGAGGCAGGCATG | |  |  |  |
|  | 03665-QF1 | | TGGCATCAAGACAGACATGTA | | 57 | 147 |  |
|  | 03665-QR1 | | ACAACGCGAGCGATGTCGAT | |  |  |  |
|  | 03393-QF1 | | AAAGGTGTTTGAGAGCGGAC | | 58 | 102 |  |
|  | 03393-QR1 | | ATCTCCCTCTCCACAACAGC | |  |  |  |
|  | 00183-QF1 | | TATGTCCCTGGCGGCTTTAA | | 58 | 145 |  |
|  | 00183-QR1 | | TGATCCACTCGCAGTCTTCA | |  |  |  |
|  | 04954-QF1 | | GTTGCGACGAGTTCTTGT | | 58 | 169 |  |
|  | 04954-QR1 | | ACCATCACCTTGCCCATA | |  |  |  |
|  | 00189-QF1 | | ACTGCTCAACGTTACGATGTTCTC | | 56 | 71 |  |
|  | 00189-QR1 | | GCCACCAGGAAGGGATAGTTG | |  |  |  |
