# Supplementary material for: A hypovirulence-associated capsidless bi-segmented ssRNA mycovirus enhances melanin and microsclerotial production in a vascular phytopathogenic fungus
Source: PLoS Pathog. 2025 Aug 11;21(8):e1013348. doi: 10.1371/journal.ppat.1013348 (PMC12360652; doi:10.1371/journal.ppat.1013348)
Supplement: S4 Table — (DOCX) [file ppat.1013348.s014.docx]

## Table S4. Primers used for vector construction in this study.

| **Function** | | **Primer** | | **Sequence (5' to 3')** | | **Tm (℃)** | | **Product (bp)** | | |
| --- | --- | --- | --- | --- | --- | --- | --- | --- | --- | --- |
| Gene replacement | | pUC-3665D F1 | | Tctttctagaggatccccggg  GAAGGCACCGTGGACAAGC | | 58 | | 1060 |  |  |
|  |  | pUC-3665D R1 | | Aattcgagctcggtacccggg  TCTTGCCCCCAGCCATCT | |  |  |  |  |  |
|  |  | pUC-3665U F1 | | Gccagtgccaagcttgcatgc  AAGGGTATGGGCCATTGGTAA | | 58 | | 1068 |  |  |
|  |  | pUC-3665U R1 | | Tctgtcgacctgcaggcatgc  TGGTGAGAGAGGATGAGATTGCC | |  |  |  |  |  |
|  |  | pUC-3393U F1 | | Gccagtgccaagcttgcatgc  ACATCTTCATCCCTACCGCAGA | | 58 | | 1138 |  |  |
|  |  | pUC-3393U R1 | | Tctgtcgacctgcaggcatgc  TGACCACTGTCACCTTTTGTATGG | |  |  |  |  |  |
|  |  | pUC-3393D F1 | | Tctttctagaggatccccggg  GGCGACACGTTTGAAAGATGG | | 58 | | 1655 |  |  |
|  |  | pUC-3393D R1 | | Aattcgagctcggtacccggg  CCCTCGACTCCTGACTAAACAGTAT | |  |  |  |  |  |
|  |  | 03393UUF | | ACGAAGACCCTCATGACAGT | |  | |  |  |  |
|  |  | 03393DDR | | ATGAGGTCAAGTTTGCTTCATG | |  | |  |  |  |
|  |  | 03665UUF | | TGACACATATGAGTTGTGACGA | |  | |  |  |  |
|  |  | 03665DDR | | GTCTACCCATCCTGCAGG | |  | |  |  |  |
|  |  | hp-F | | AGAAGATGATATTGAAGGAGCAC | |  | |  |  |  |
|  |  | ph-R | | AAGAAGGATTACCTCTAAACAAG | |  | |  |  |  |
|  |  | HYG-F | | GCGAAGAATCTCGTGCTTTC | | 56 | | 528 |  |  |
|  |  | HYG-R | | CCGTCAGGACATTGTTGGA | |  |  |  |  |  |
| Y2H vector  construction | | BDV2R2F | | Cggggatccgtcgacctgca  ATGACTAGTTATAGTCTTGCCCCT | | 58 | | 1600 |  |  |
|  |  | BDV2R2R | | Ctagttatgcggccgctgca  CTAGGCTTCCTTTGATGCC | |  |  |  |  |  |
|  |  | AD03393F | | Acccgggtgggcatcgatacgg  AATGGCAGCAGCACCC | | 58 | | 642 |  |  |
|  |  | AD03393R | | Catctgcagctcgagctcgat  GTCAACTGGCCGCCGC | |  |  |  |  |  |
|  |  | AD03665F | | Acccgggtgggcatcgatacgg  AATGCCTGGCGCCAC | | 58 | | 894 |  |  |
|  |  | AD03665R | | Catctgcagctcgagctcgatg  TTACATGCAAGCGGCACCG | |  |  |  |  |  |
|  |  | AD04551F | | Acccgggtgggcatcgatacgg  AATGCTTTCTCTCCAGACCG | | 58 | | 804 |  |  |
|  |  | AD04551R | | Catctgcagctcgagctcgatg  CTACGAGCAGTGAACAAAGCC | |  |  |  |  |  |

|  | | | |
| --- | --- | --- | --- |
| **Family** | **Virus name** | **Abbreviation** | **Accession** |
| Established ormycoviruses | Erysiphe lesion-associated ormycovirus 1 | ElaOMV1 | OM272927 |
|  | Erysiphe lesion-associated ormycovirus 2 | ElaOMV2 | OM272931 |
|  | Erysiphe lesion-associated ormycovirus 3 | ElaOMV3 | OM363731 |
|  | Erysiphe lesion associated ormycovirus 4 | ElaOMV4 | OM272933 |
|  | Downy mildew lesion associated ormycovirus 1 | DmlaOMV1 | OM363727 |
|  | Downy mildew lesion associated ormycovirus 2 | DmlaOMV2 | OM262448 |
|  | Downy mildew lesion associated ormycovirus 3 | DmlaOMV3 | OM363729 |
|  | Downy mildew lesion associated ormycovirus 4 | DmlaOMV4 | OM272935 |
|  | Downy mildew lesion associated ormycovirus 5 | DmlaOMV5 | OM272937 |
|  | Downy mildew lesion associated ormycovirus 6 | DmlaOMV6 | OM262449 |
|  | Downy mildew lesion associated ormycovirus 7 | DmlaOMV7 | OM262450 |
|  | Starmerella bacillaris ormycovirus 1 | SbOMV1 | OM272929 |
|  | Uromyces appendiculatus ormycovirus 2 | UaOMV2 | GACI01004785.1 |
|  | Ambispora leptoticha ormycovirus 1 | AlOMV1 | GGIK01050282.1 |
|  | Uromyces appendiculatus ormycovirus 1 | UaOMV1 | GACI01002316.1 |
|  | Puccinia striiformis ormycovirus 1 | PsOMV1 | GAIR01011407.1 |
|  | Trichoderma tomentosum ormycovirus 1 | TtOV1 | WGH72997.1 |
