# Supplementary material for: A hypovirulence-associated capsidless bi-segmented ssRNA mycovirus enhances melanin and microsclerotial production in a vascular phytopathogenic fungus
Source: PLoS Pathog. 2025 Aug 11;21(8):e1013348. doi: 10.1371/journal.ppat.1013348 (PMC12360652; doi:10.1371/journal.ppat.1013348)
Supplement: S5 Table — (DOCX) [file ppat.1013348.s015.docx]

| Table S5. The list of ormycoviruses used in alignment analysis and phylogenetic analysis in this study. | | | |
| --- | --- | --- | --- |
| **Family** | **Virus name** | **Abbreviation** | **Accession** |
| ormycoviruses | Erysiphe lesion-associated ormycovirus 1 | ElaOMV1 | OM272927 |
|  | Erysiphe lesion-associated ormycovirus 2 | ElaOMV2 | OM272931 |
|  | Erysiphe lesion-associated ormycovirus 3 | ElaOMV3 | OM363731 |
|  | Erysiphe lesion associated ormycovirus 4 | ElaOMV4 | OM272933 |
|  | Downy mildew lesion associated ormycovirus 1 | DmlaOMV1 | OM363727 |
|  | Downy mildew lesion associated ormycovirus 2 | DmlaOMV2 | OM262448 |
|  | Downy mildew lesion associated ormycovirus 3 | DmlaOMV3 | OM363729 |
|  | Downy mildew lesion associated ormycovirus 4 | DmlaOMV4 | OM272935 |
|  | Downy mildew lesion associated ormycovirus 5 | DmlaOMV5 | OM272937 |
|  | Downy mildew lesion associated ormycovirus 6 | DmlaOMV6 | OM262449 |
|  | Downy mildew lesion associated ormycovirus 7 | DmlaOMV7 | OM262450 |
|  | Starmerella bacillaris ormycovirus 1 | SbOMV1 | OM272929 |
|  | Uromyces appendiculatus ormycovirus 2 | UaOMV2 | GACI01004785.1 |
|  | Ambispora leptoticha ormycovirus 1 | AlOMV1 | GGIK01050282.1 |
|  | Uromyces appendiculatus ormycovirus 1 | UaOMV1 | GACI01002316.1 |
|  | Puccinia striiformis ormycovirus 1 | PsOMV1 | GAIR01011407.1 |
|  | Trichoderma tomentosum ormycovirus 1 | TtOV1 | WGH72997.1 |
